# Phenotypic Diversity in Maize Landraces: A Systematic Review of Global Patterns, Methodological Approaches, and Implications for Breeding

**DOI:** 10.3390/genes17040413

**Published:** 2026-03-31

**Authors:** Suwilanji Nanyangwe, Arsenio Daniel Ndeve, Pedro Fato, Paulino Munisse, Kolawole Peter Oladiran, Constantino Francisco Lhamine, Mable Chebichii Kipkoech

**Affiliations:** 1 Department of Crop Production, Faculty of Agronomy and Forest Engineering, Eduardo Mondlane University, Maputo P.O. Box 257, Mozambique; ndevegod@gmail.com (A.D.N.); oladirankolawole5@gmail.com (K.P.O.); bycosta.francisco@gmail.com (C.F.L.); mablekipkoech13@gmail.com (M.C.K.); 2 Centre of Excellence in Agri-Food Systems and Nutrition (CE-AFSN), Eduardo Mondlane University, Praca 25 de Junho Edificio da Reitoria 5° Andar, Maputo P.O. Box 257, Mozambique; 3 Zambia Agriculture Research Institute, Mount Makulu Research Station, Chilanga 10101, Zambia; 4 Instituto de Investigação Agrária de Moçambique (IIAM), Maputo P.O. Box 3558, Mozambique; pmunisse@gmail.com

**Keywords:** maize landraces, genetic diversity, phenotypic diversity, agro-morphological traits, plant breeding, genetic resources, adaptation

## Abstract

**Background/Objective:** Maize (*Zea mays* L.) is a globally important cereal crop widely cultivated for food, feed, fodder, biofuel production, and various industrial applications. Maize landraces represent a valuable source of genetic diversity that supports adaptationand resilience across diverse agroecological environments. However, evidence on phenotypic diversity based on agro-morphological traits in these landraces remains fragmented across regions and varying analytical approaches. This review synthesized global evidence on phenotypic variation, heritability patterns, experimental designs, statistical methods, and the extent of integration between phenotypic and molecular data. **Methods:** A systematic literature search was conducted in GoogleScholar, ScienceDirect, PubMed and AGRIS for studies published between 2000 and 2025 evaluating phenotypic diversity in maize landraces. The review followed PRISMA 2020 guidelines, and f50 studies from 30 countries met the eligibility criteria. **Results:** Substantial and structured phenotypic diversity was consistently reported across studies, with flowering time, plant architecture, and ear and kernel traits emerging as major contributors to landrace differentiation. Traits with moderate to high heritability were mainly morphological and phenological, suggesting relative genetic control and potential suitability for phenotypic selection. In contrast, grain yield showed greater environmental sensitivity and variable heritability, reflecting complex inheritance and genotype × environment interactions. Although molecular markers were incorporated in a some studies, integrative analyses linking phenotypic and genetic data remained limited. **Conclusions:** Phenotypic evaluation remains a reliable approach for characterizing maize landrace diversity. However, standardized methodologies, greater integration with molecular data and cross-environment validation are needed to strengthen inference and utilization in breeding and conservation. The review also provides recommendations for improving agro-morphological assessment in maize landraces.

## 1. Introduction

Maize (*Zea mays* L.) is one of the most important cereal crops worldwide. It serves as a staple food for millions of people, a major component of animal feed, and a raw material for numerous industrial applications [1,2]. Owing to its high productivity, wide adaptability, and diverse end uses, maize contributes substantially to food security, rural livelihoods, and agricultural economies in many regions of the world [3]. Its cultivation across diverse agroecological zones has generated substantial genetic and phenotypic diversity within the species, which contributes to adaptation under variable environments and supports resilience to climate variability, low-input farming systems, and emerging biotic and abiotic stresses [4,5]. However, increasing global demand for food and feed, combined with the growing challenges of climate change, pests, and diseases, continues to place pressure on maize production systems, highlighting the need for the development of resilient and well-adapted varieties.

Crop improvement efforts rely on the availability and effective use of genetic resources that harbor traits for adaptation, yield stability, and stress tolerance. Among these resources, maize landraces represent an important reservoir of genetic diversity, often expressed as wide variation in agro-morphological traits [6]. Landraces are traditional open-pollinated populations shaped through long-term natural and farmer-mediated selection under diverse environmental conditions [7,8]. As heterogeneous populations, they retain substantial within and among population variation in key agro-morphological traits, including flowering time, plant and ear architecture, kernel characteristics, and yield components, as well as variation in nutritional composition and end-use quality traits relevant for food processing and industrial utilization [9,10]. This diversity arises from cross-pollination, introgression from different germplasm sources, and long-term adaptation to diverse local environments [11]. Consequently, maize landraces provide breeders with valuable alleles that can be exploited to improve adaptation, yield stability, and resilience to biotic and abiotic stresses [12,13,14]. Despite their importance, maize landraces are increasingly threatened by genetic erosion as modern hybrids replace traditional varieties, narrowing the germplasm pool available for crop improvement [15,16]. Systematic characterization and documentation of landrace diversity are therefore essential to support their conservation and effective utilization in breeding programs.

In recent years, research on maize landraces has expanded alongside methodological advances, including the integration of molecular tools with phenotypic assessments to improve understanding of genotype–phenotype relationships [17,18,19]. The use of molecular markers and quantitative trait loci (QTLs) associated with key traits has enhanced the identification of germplasm with favourable alleles [20,21]. Because molecular markers are largely unaffected by environmental conditions, they provide stable genetic information that facilitates marker-assisted selection across diverse populations [22,23]. Although molecular and genomic studies have demonstrated substantial genetic variation in maize landraces, field-based phenotypic evaluation remains essential, as trait expression is influenced by environmental conditions and genotype × environment interactions [24,25]. Evaluation of agro-morphological traits, therefore, provides a practical means of characterizing maize diversity under field conditions, offering an indirect measure of underlying genetic variation. Such evaluations support the identification of genotypes with desirable and heritable characteristics that can be incorporated into breeding programs to develop varieties with improved performance and farmer acceptance across contrasting production environments [26,27,28].

However, methodological differences among phenotypic diversity studies have limited comparability and synthesis across regions, largely due to differences in experimental design, trait selection, statistical analyses, and reporting practices. In particular, variation in replication level and experimental structure strongly influences the robustness of phenotypic inference. While replicated and well-structured trials tend to produce reliable and comparable outcomes, studies conducted under unreplicated or weakly structured designs often suffer from reduced statistical power and limited inferential strength [29,30,31]. In addition, inconsistent use of trait descriptors and incomplete reporting of environmental metadata further constrain reproducibility and cross-study synthesis. Moreover, despite the increase in the number of studies on maize landrace diversity based on agro-morphological traits, there is currently limited systematic synthesis evaluating experimental design, trait selection, statistical workflows, and reporting practices within a global evidence base. Existing reviews, including [15,32,33,34], are largely narrative in nature and focus on the importance, conservation, and utilization of maize genetic resources, but do not explicitly assess methodological patterns that influence the interpretation, comparability, and practical utility of phenotypic diversity estimates derived from field-based studies. This indicates the need for a systematic review to evaluate methodological variation and synthesize evidence from field-based agro-morphological studies of maize landrace.

To address these methodological gaps, the objectives of the review were to: (i) identify and synthesize published studies reporting phenotypic diversity in maize landraces based on agro-morphological traits; (ii) evaluate experimental designs, statistical methods and reporting practices applied across studies and identify methodological inconsistencies affecting phenotypic inference and (iii) determine the agro-morphological traits most frequently assessed and their relative contribution to reported diversity patterns. By consolidating the available evidence, this review provides guidance for harnessing maize landrace diversity in breeding and future research.

## 2. Materials and Methods

### 2.1. Scope and Framework

This review adopted a global scope to capture methodological diversity and trait use across the wide range of agroecological environments in which maize landraces are cultivated. Studies published between 2000 and 2025 were included to reflect both recent advances and long-term trends. The focus was on maize landraces, as they harbour high genetic and phenotypic diversity, which is valuable for breeding and conservation, and remain central to smallholder production systems. Studies that included improved varieties were considered eligible only when they used them as comparators. Eligibility criteria were defined using the PICOTS framework which is widely used for structuring research questions and eligibility criteria in systematic reviews [35,36]: population (maize landraces), intervention (field-based phenotypic evaluation), comparators (checks when used), outcomes (diversity based on agro-morphological traits), timing (publication period defined above), and setting (field trials).

### 2.2. Protocol Registration and Reporting Transparency

This systematic review was conducted and reported in accordance with the Preferred Reporting Items for Systematic Reviews and Meta-Analyses (PRISMA) 2020 guidelines [37]. The review protocol was registered in the Open Science Framework (OSF; https://doi.org/10.17605/OSF.IO/YHBN7) before data extraction to enhance transparency, ensure reproducibility and minimize the risk of post hoc changes to the review process.

### 2.3. Information Sources

A systematic literature search was conducted in September 2025 to identify relevant field-based studies on maize landrace phenotypic diversity. The databases searched included Google Scholar (searched on 9 September 2025), ScienceDirect (9 September 2025), PubMed (20 September 2025), and AGRIS (20 September 2025). In addition, the reference lists of included studies were screened to capture any additional relevant publications. No database updates were performed after September 2025, and this cut-off was selected to ensure consistent screening and extraction within the defined review timeline.

### 2.4. Search Strategy

A structured search strategy was developed to retrieve relevant studies on phenotypic diversity in maize landraces across major scientific databases. Search strings combined terms related to the crop, germplasm type, and diversity assessment using Boolean operators. The following search string was applied across databases, with minor adjustments depending on database search syntax: *(maize OR “Zea mays”) AND (landrace OR “local varieties” OR “traditional varieties”) AND (“phenotypic diversity” OR “agro-morphological characterization” OR “genetic diversity”)*. The term ‘corn’ was explored during preliminary searches; however, it retrieved a large number of irrelevant records related to sweet corn physiology, food processing, and non-landrace studies. Therefore, the term ‘maize’, the standard scientific name for *Z. mays*, was retained in the final search strategy to improve specificity.

In Google Scholar, the search was conducted using Publish or Perish version 8 [38,39], which enables systematic retrieval and export of search results. The search was limited to the first 1000 retrievable records, representing the maximum number of results that can be exported through this platform. Although additional records may exist beyond this threshold, Google Scholar ranks results using a relevance-based algorithm, and limiting retrieval to the first 1000 records captures the highest-ranked and most relevant studies while maintaining a systematic and reproducible search process [40]. Retrieved records were exported as RIS files. In ScienceDirect, filters were applied to restrict results to research articles within relevant subject areas, and records were exported in RIS format. In PubMed and AGRIS, filters for publication date, English language, and full-text availability were applied, and the retrieved records were exported in CSV and RIS formats, respectively. Despite limiting the search to English-language publications, exploratory searches using Spanish, Portuguese, and Chinese terms were conducted to assess the potential influence of this restriction.

### 2.5. Selection Process

The retrieved records were screened in two sequential stages: initial title and abstract screening followed by full-text assessment to determine eligibility and relevance to the review objectives. Screening was conducted using Microsoft Excel 2019 (Microsoft Corporation, Redmond, WA, USA) to organize and track records. Two reviewers independently screened titles and abstracts according to the predefined inclusion and exclusion criteria, and the same reviewers assessed full-text articles to confirm eligibility. Disagreements at either stage were resolved through discussion and consensus, and where necessary, a third reviewer was consulted to reach a final decision. No automation tools were used during the title-abstract screening or study selection process. Only studies that met the inclusion criteria after full-text assessment were retained for inclusion in the review.

### 2.6. Eligibility Criteria

Eligibility criteria defined using the PICOTS framework described in the scope were applied during both title-abstract screening and full-text review.

#### 2.6.1. Inclusion Criteria

Studies were included if they were peer-reviewed articles reporting field-based phenotypic evaluation of maize landraces under experimental or on-farm conditions, assessed agro-morphological traits, and were available as full-text articles in English. Studies incorporating improved varieties or hybrids were eligible only when these were used as comparator cultivars (checks) alongside landraces. In the reviewed studies, landraces and improved varieties were clearly distinguished based on the descriptions provided by the original authors and verified during data extraction. Improved varieties or hybrids were therefore treated as reference checks for comparison, while the interpretation of diversity patterns primarily focused on variation among landraces.

#### 2.6.2. Exclusion Criteria

Studies were excluded if they focused exclusively on commercial hybrids or inbred lines, relied solely on molecular data without accompanying field-based phenotypic evaluation, or lacked sufficient methodological detail for comparative synthesis. Review articles, opinion papers, conference abstracts, and studies unrelated to maize were also excluded. Grey literature (for instance, theses and technical reports) was screened but excluded to maintain consistency in methodological rigor, reporting standards, and long-term accessibility of the evidence base.

### 2.7. Data Collection Process

Data were extracted using a structured Excel sheet that captured bibliographic information, study characteristics (location, environment, trial type), experimental design (genotypes, checks, traits, design, replications, plot size, spacing, plant density, sample size), trait descriptors, measurement units, statistical methods, and key findings. The extraction template and extracted dataset are available in the Open Science Framework repository at https://doi.org/10.17605/OSF.IO/YHBN7. Data extraction was performed by one reviewer using predefined categories and was subsequently cross-checked against the original studies by a second reviewer for accuracy and completeness. Any discrepancies identified during cross-checking were resolved through discussion and verification against the source publications. No automation tools were used during the data extraction process.

### 2.8. Data Items

The primary outcomes included agro-morphological traits used to assess phenotypic diversity in maize landraces, such as flowering time, plant architecture, ear and kernel characteristics, yield-related traits, and associated measures of variability and heritability, where reported. All results related to these outcome domains were considered during data extraction, including descriptive statistics, inferential analyses, and multivariate outputs reported in the original studies. In addition to trait outcomes, contextual and methodological variables were recorded to support synthesis and interpretation of findings across studies. These variables provided information on study setting, experimental characteristics, and analytical approaches, enabling structured comparison and synthesis across studies.

#### Handling of Missing Data

When required information was not explicitly reported in the main text, Supplementary Materials associated with the publication were examined. Where experimental details or trait information could not be retrieved from either the main article or Supplementary Files, missing information was recorded as “not reported.” Assumptions were only made for plant density, which was calculated from reported spacing regimes when not explicitly provided. The completed dataset served as the basis for descriptive summaries, synthesis, and further analysis of the included studies.

### 2.9. Risk of Bias Assessment

Risk of bias was assessed across four methodological domains relevant to field-based phenotypic diversity studies: study design, replication, trait measurement rigor, and statistical analysis appropriateness. Because the included studies were field-based agronomic experiments rather than randomized intervention trials, standard risk-of-bias tools developed for clinical studies such as RoB 2 were not directly applicable. Therefore, a domain-based framework adapted from general systematic review guidance [41] was used to evaluate methodological factors that could influence the reliability and interpretability of reported diversity estimates. To ensure consistent evaluation across studies, predefined criteria were used to assign risk-of-bias ratings within each domain as low, unclear (some concerns), or high. Assessments were visualized using the RoBVis tool [42] with the generic dataset option, which allows visualization of customized risk-of-bias domains across studies.

Bias domains and assessment criteria

**Bias due to Study Design**

This domain evaluated whether studies used clearly defined experimental designs appropriate for field-based agronomic trials, such as randomized complete block designs (RCBD), lattice designs, or augmented designs.

- Low risk: Appropriate experimental design clearly reported.
- Unclear risk: Experimental design not reported or insufficiently described.
- High risk: Experimental layout unclear or clearly inappropriate for field trials (e.g., characterization trials conducted without replication, accessions sown in single-row plots and evaluated without proper experimental structure).

**Bias due to Replication**

This domain assessed the adequacy of experimental replication reported in the studies.

- Low risk: At least two replications clearly reported.
- Unclear risk: Replication not reported or insufficiently described.
- High risk: Replication explicitly stated as absent (e.g., single unreplicated plots).

**Bias due to Trait Measurement Rigor**

This domain evaluated the clarity and rigor of agro-morphological trait measurement procedures.

- Low risk: Traits measured using recognized standard descriptors (e.g., CIMMYT, IBPGR) with clearly defined units and measurement procedures.
- Unclear risk: Traits reported, but descriptor systems not cited or measurement procedures partly unclear.
- High risk: Traits poorly defined or ambiguously described, lacking clear definitions, units, or measurement procedures.

**Bias due to Appropriateness of statistical Analysis**

This domain assessed whether appropriate statistical methods for evaluating phenotypic diversity were applied and clearly reported.

- Low risk: Appropriate statistical methods applied and clearly reported (e.g., ANOVA, PCA, cluster analysis, diversity indices).
- Unclear risk: Statistical methods mentioned but insufficiently described (e.g., lack of detail on model specification, assumptions, or analytical procedures)
- High risk: Statistical methods inappropriate for the study objective or key analyses missing (e.g., absence of inferential or multivariate analyses where required).

An overall risk-of-bias judgement was assigned for each study based on the domain-level assessments following general systematic review guidance [41]. Studies were classified as low risk of bias when all domains were rated as low risk. Studies were judged unclear (some concerns) when at least one domain was rated as unclear risk, but none were rated as high risk. Studies were classified as high risk of bias when at least one domain was rated as high risk, or when concerns across multiple domains substantially reduced confidence in the reported results.

### 2.10. Effect Measures

This review did not estimate pooled effect sizes; instead, effect measures were summarized descriptively using reported measures of phenotypic variation, heritability, and trait contribution to diversity across studies. Meta-analysis was not performed due to substantial methodological heterogeneity among studies, including differences in experimental design, trait selection, and analytical approaches.

### 2.11. Synthesis Methods

Studies were grouped for synthesis based on study characteristics, experimental design, traits assessed, analysis methods and key findings. This approach enabled a structured presentation of results and facilitated comparison across studies. Results were summarized descriptively using frequency tables and graphical displays (bar charts, pie charts, and RoBVis plots). Narrative and tabular synthesis were used to compare findings across studies. Variability was explored by examining differences among studies conducted across diverse agroecological zones and countries to identify global methodological patterns and the traits most frequently associated with genetic differentiation among maize landraces. Heterogeneity, sensitivity of findings, reporting bias, and certainty of evidence were examined qualitatively using predefined criteria. Heterogeneity was assessed by comparing studies based on population size, experimental design, replication, and number of evaluation environments, while sensitivity, reporting bias, and certainty of evidence were evaluated based on consistency of findings, completeness of reporting, and overall methodological quality.

## 3. Results

### 3.1. Study Selection

A total of 1905 records were retrieved from Google Scholar (n = 1000), ScienceDirect (n = 674), PubMed (n = 179), and AGRIS (n = 52). A total of 1769 unique studies remained for screening after removal of 136 duplicate records using Microsoft Excel. The relatively small number of duplicates reflects differences in database coverage and indexing practices, particularly because Google Scholar retrieves a broader range of source types that do not always overlap directly with records indexed in other databases [40]. Preliminary title and abstract screening excluded 1696 records that did not meet the eligibility criteria.

**Figure 1:**
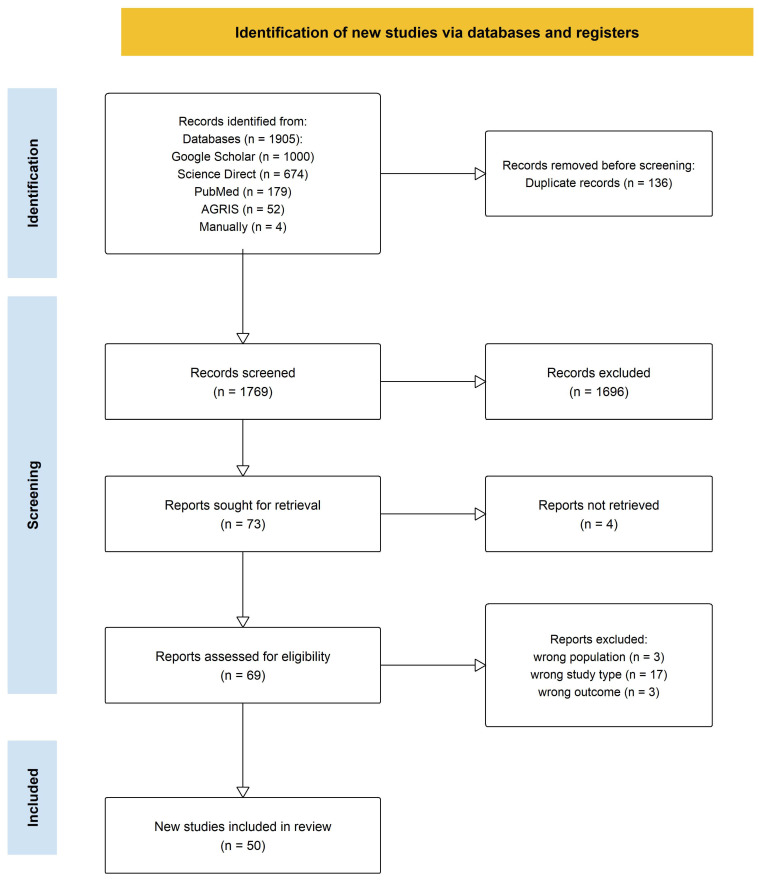
PRISMA flow diagram showing the identification, screening, eligibility, and inclusion of studies; n = number of studies. Source: [43].

The full texts of 73 studies were sought for retrieval, of which four could not be accessed due to paywall restrictions or limitations in full-text availability. Consequently, 69 full-text articles were assessed for eligibility, and 23 studies were further excluded for the following reasons: inappropriate population (including hybrids, inbreds, or double haploids) (n = 3); inappropriate study type, including molecular-only analyses without field-based phenotypic evaluation (n = 15) and review articles or theses (n = 2); inappropriate outcomes, specifically biotic trait studies outside the agro-morphological trait scope (n = 2); and kernel biochemical composition studies (n = 1). In total, 46 studies were included from database searches, and four additional studies were identified through reference lists, resulting in a final set of 50 studies. Of these, 38 were sourced from Google Scholar, one from ScienceDirect, five from PubMed, and two from AGRIS. The study selection process is illustrated in the PRISMA 2020 flow diagram (Figure 1).

### 3.2. Excluded Studies

Studies excluded after full-text assessment fell into several recurring categories. A small number of studies were excluded due to inappropriate study populations, as they focused on hybrids, inbred lines, or double haploid materials rather than maize landraces, which were the target population of this review [44,45,46]. Although these studies provided valuable insights into maize genetics, their focus on improved or highly controlled genetic materials rendered them unsuitable for addressing phenotypic diversity among landraces.

A larger proportion of exclusions resulted from inappropriate study types, particularly studies that relied exclusively on molecular marker analyses without accompanying field-based phenotypic evaluation for instance [24,47,48,49,50,51,52,53,54], While these studies contributed important information on genetic structure and diversity, they did not align with the objectives of this review, which emphasized agro-morphological characterization under field conditions. In addition, review articles such as [55] were excluded because they did not present original experimental data suitable for synthesis, and academic theses such as [56] were excluded because they are not peer-reviewed publications and fell outside the defined scope of this review, which was restricted to peer-reviewed journal articles. Some studies were also excluded due to the inaccessibility of the full text [57,58,59,60]. Finally, a small number of studies were excluded due to inappropriate outcomes, including investigations centered on biotic stress traits outside the agro-morphological trait scope and studies focusing on kernel biochemical composition rather than phenotypic diversity relevant to agronomic performance [61,62,63].

### 3.3. Characteristics of the Selected Studies

Across the 50 reviewed studies, heterogeneity was observed in experimental design, trait coverage, and the number of genotypes evaluated. Most studies were field-based experiments assessing multiple agro-morphological traits across diverse agroecological environments. Commonly applied statistical analyses included ANOVA, PCA, cluster analysis, and correlation methods. (Table 1) summarizes key characteristics of the individual studies, including numbers of genotypes, experimental designs, traits assessed, statistical analyses, and principal findings related to phenotypic diversity. Detailed study-level information is provided in Supplementary Table S1.

All included studies employed experimental approaches to assess agro-morphological diversity in maize landraces. 49 studies were based solely on experimental data, while one combined experimental and secondary data sources. Field trials alone were conducted in 34 studies, whereas 15 studies combined field phenotyping with molecular characterization. The number of genotypes evaluated ranged from 9 to 588 (median = 53), and studies assessed between 6 and 41 traits (average = 18). Replication was commonly applied, with most studies using two or three replications. Improved varieties or hybrids were included as checks in 27 studies.

Experimental layout and measurement protocols varied across studies. Plot lengths typically ranged from 3 to 6 m, with one- or two-row plots most common, and sampling was generally based on 5–10 plants per plot. Trait assessment protocols were often based on CIMMYT-IBPGR descriptor developed in (1991), which were explicitly reported in 27 studies. However, descriptor systems were not specified in 19 studies, while a small number referenced alternative descriptor frameworks. Row spacing was most commonly 0.75 m with intra-row spacing of 0.20–0.40 m, although spacing information was not consistently reported. Planting densities ranged from approximately 28,000 to 166,667 plants ha^−1^, with 44,444 and 53,333 plants ha^−1^ most frequently reported. Aggregated summaries of study characteristics are presented in Table 2, while detailed study-level information is provided in Supplementary Table S2.

### 3.4. Geographic Distribution of Included Studies

**Figure 2:**
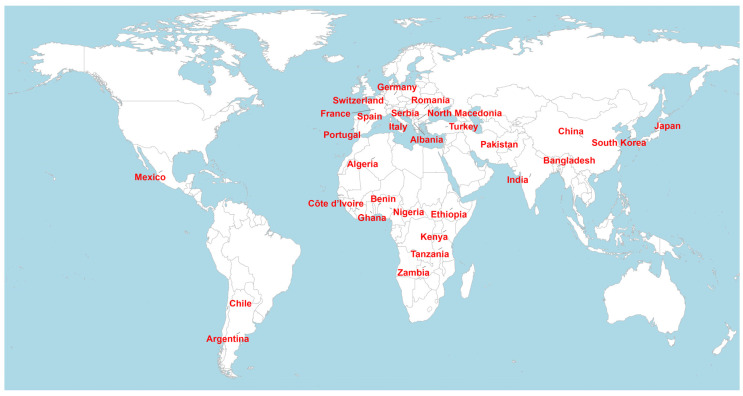
Geographic distribution of countries represented in field-based studies on phenotypic diversity in maize landraces included in the systematic review.

The distribution of studies included in this review reflects the global pattern of published field-based research on phenotypic diversity in maize landraces (Figure 2). The 50 eligible studies were conducted across 30 countries spanning Africa, the Americas, Asia, and Europe, with uneven representation among regions and countries. India, Serbia, and Mexico contributed the highest number of publications, while several European and African countries were represented by a smaller number of studies (Supplementary Table S3). The reviewed studies were conducted under diverse agro-ecological conditions and experimental settings, including different locations, seasons, and management practices, to assess agro-morphological variation among maize landraces. In contrast, several regions, particularly parts of East and Southern Africa and the Americas, were represented by relatively few publications, highlighting geographic imbalances in the existing literature.

### 3.5. Experimental and Analytical Characteristics of Studies

#### 3.5.1. Experimental Designs Used in the Studies

In the 50 studies included in this review, a range of experimental designs was used to evaluate phenotypic diversity in maize landraces (Table 3). The randomized complete block design (RCBD) was the most frequently applied (23 studies), followed by alpha-lattice, simple lattice, and augmented block designs, each used in six studies. Less conventional or unreported designs were used in nine studies, mainly in exploratory or characterization trials. The distribution of experimental designs differed across studies with varying numbers of genotypes and levels of field variability.

#### 3.5.2. Key Agro-Morphological Traits Assessed

**Figure 3:**
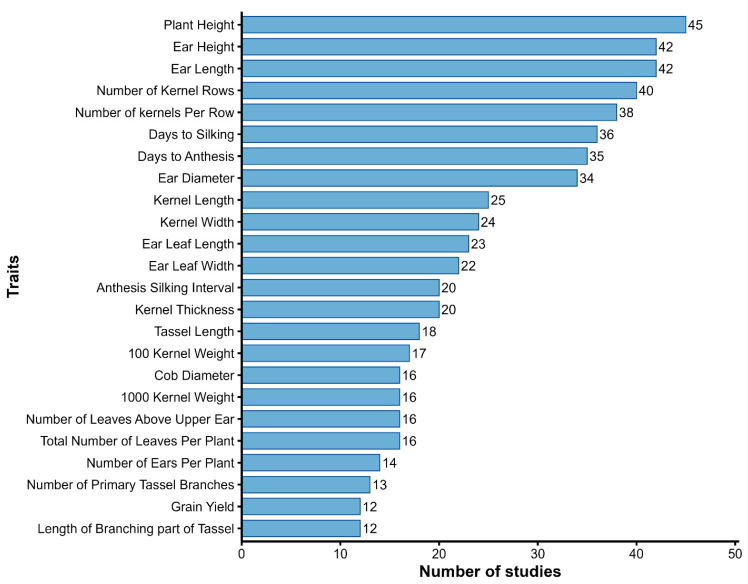
Frequency of key agro-morphological traits assessed, highlighting traits commonly used to infer underlying genetic variation among maize landraces.

The included studies assessed a wide range of agro-morphological traits to characterize phenotypic diversity in maize landraces, with particular emphasis on plant architecture, flowering time, ear and kernel traits, and yield-related characteristics. In total, 961 trait mentions were recorded, representing 212 unique agro-morphological traits across the reviewed studies. The most frequently measured traits were plant height (45 studies), ear height (42), ear length (42), number of kernel rows (40), number of kernels per row (38), days to silking (36), days to anthesis (35), and ear diameter (34), highlighting their central role in adaptation and productivity (Figure 3). Kernel size traits, including kernel length (25) and kernel width (24), were also commonly evaluated due to their relevance to grain quality.

Across studies, a consistent pattern was observed in the selection of traits used to assess phenotypic diversity. Flowering time, plant height, ear height, ear length, ear diameter, and kernel traits were repeatedly measured across diverse experimental designs and environments. In contrast, tassel traits, leaf architectural traits, and physiological traits were assessed less frequently.

**Figure 4:**
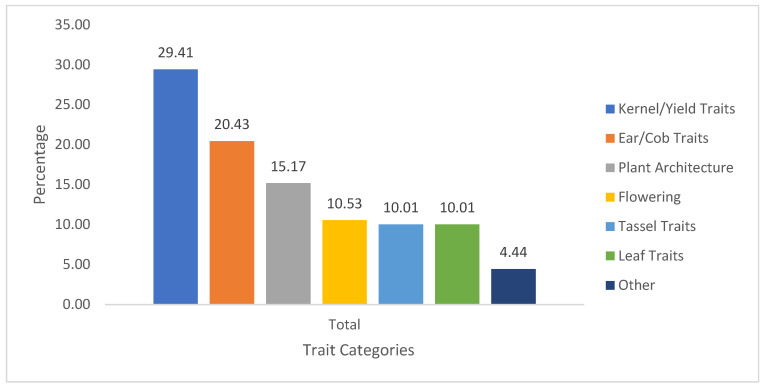
Percentage distribution of agro-morphological trait categories assessed in maize landrace diversity studies.

When traits were grouped by category, kernel and yield traits accounted for the largest share (29.41%), emphasizing productivity-related measures, while ear and cob traits represented 20.43%, describing ear morphology. Flowering traits (10.53%) and plant architectural traits (15.17%) were reported at similar frequencies, highlighting their importance for adaptation and growth characterization. Leaf and tassel traits were each represented at 10.01%, while other traits accounted for 4.44% (Figure 4). The ‘Other’ category comprised traits such as disease and pest scores, nutrient content, growth rate, and photosynthetic activity, which were reported only in a subset of the studies included in this synthesis. Overall, the distribution of trait categories indicates that phenotypic diversity studies of maize landraces commonly focus on plant stature, reproductive timing, and ear and kernel characteristics.

#### 3.5.3. Statistical Analysis Methods Applied

**Figure 5:**
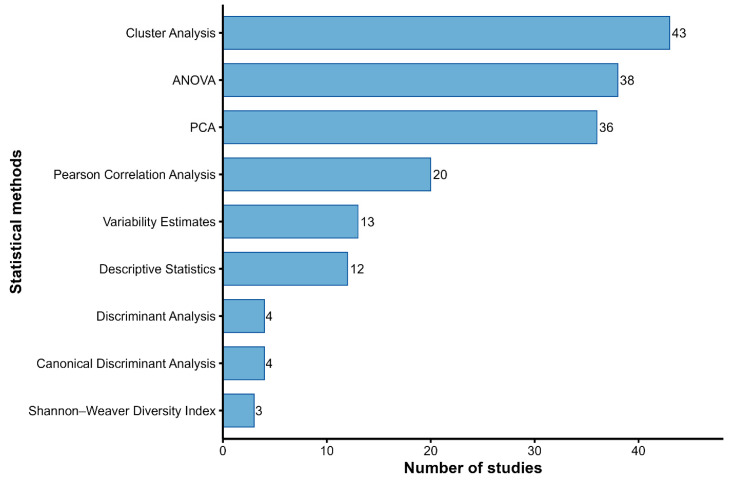
Frequency of the top 10 statistical analysis methods used in studies on phenotypic diversity of maize landraces.

To analyze variation in the measured traits and examine patterns of phenotypic diversity among landraces, the reviewed studies employed a range of statistical and multivariate analytical methods. Cluster analysis (43), analysis of variance (ANOVA) (38), and principal component analysis (PCA) (36) were the most frequently reported methods (Figure 5). Together, these formed the core analytical framework, with ANOVA used to test for significant trait variation, PCA to reduce data dimensionality and identify traits contributing most to variation, and cluster analysis to group landraces based on similarity. Moderately used approaches included Pearson correlation (20), which helped reveal trait associations, as well as variability estimates (13) and descriptive statistics (12), which provided measures of trait spread and basic summaries. Less common but still notable were discriminant and canonical discriminant analyses (4 each), used for distinguishing among groups of landraces, and diversity indices such as the Shannon-Weaver index (3) were also applied in some studies to quantify population-level variability. Advanced methods such as mixed models, Mantel tests, Mahalanobis D^2^, and stability analyses were applied mainly in multi-environment trials for a few studies.

### 3.6. Risk of Bias in Studies

**Figure 6:**
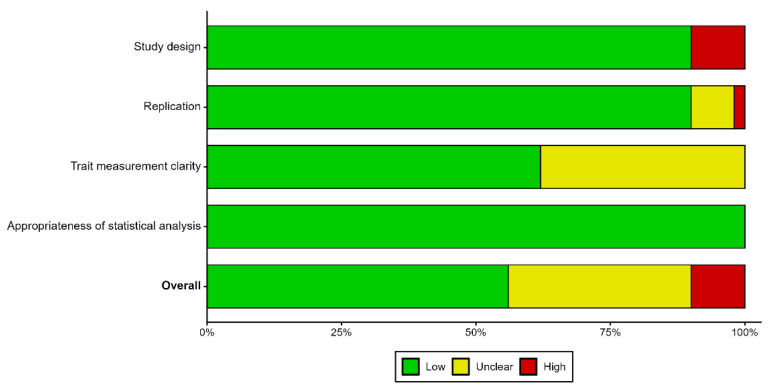
Summary bar plot of risk-of-bias assessments across domains for included studies.

Overall, most studies were rated as low risk across the assessed domains, reflecting generally acceptable methodological quality. Low-risk ratings were most prominent for appropriateness of statistical analysis, where nearly all studies were classified as low risk, followed by study design and replication, which also showed a high proportion of low-risk ratings with only a small fraction of studies categorized as high or unclear risk. In contrast, trait measurement clarity showed greater uncertainty, with a substantial proportion of studies rated as unclear, reflecting incomplete reporting of descriptor systems and measurement procedures. Across domains, high-risk ratings were relatively limited and were mainly associated with a lack of replication or weaknesses in study design. These patterns are illustrated in the summary bar plot (Figure 6), while the traffic-light plot (Figure S1) presents domain-level risk-of-bias judgments for the 50 included studies.

## 4. Results of Syntheses

### 4.1. Univariate Analysis of Phenotypic Traits Across Studies

#### 4.1.1. Analysis of Variance (ANOVA)

Across the reviewed studies, univariate statistical analyses based on ANOVA consistently detected significant phenotypic variation among maize landraces across a wide range of experimental designs. Structured designs such as randomized complete block and lattice designs, which incorporate replication of genotypes and blocking, enabled more reliable partitioning of variation into genetic and environmental components, including effects of locations, and seasons [82,101,108]. These studies frequently reported significant genotype, environment, and genotype × environment interaction effects. In contrast, augmented and unreplicated designs, while still reporting significant differences among accessions [77,84,93,96], provided limited replication for test entries. Across studies, significant differences were most frequently observed for key agro-morphological traits such as plant height, ear height, flowering time, ear length, kernel number, and grain yield, confirming substantial phenotypic differentiation among landrace collections [72,74,86,88,90]. Occasional non-significant results for traits such as grain yield or ears per plant were also reported [69,72,80,107]. Overall, a consistent cross-study pattern of significant phenotypic differentiation among maize landraces was observed for most agro-morphological traits.

#### 4.1.2. Coefficient of Variation (CV)

The magnitude of this variability, as reflected by the CV, varied across studies and methodological approaches. CV reporting could be grouped into two main categories: (i) experimental CV derived from ANOVA and (ii) phenotypic CV based on descriptive statistics. Experimental CV, representing residual variation and thus experimental precision, was reported in studies employing structured designs such as lattice and RCBD and was typically presented alongside ANOVA outputs [74,80,82,83,88,104,108]. In some multi-environment trials, experimental CV was reported across environments, providing a broader assessment of trial precision [100,110]. In contrast, phenotypic CV derived from descriptive statistics reflected overall variability among genotypes and was reported across studies using augmented, lattice, and RCBDs [69,84,86,91,92,93], Across studies, CV values varied widely, with more moderate and consistent values generally reported in structured and replicated designs [70,81,108] and higher or more variable values observed in augmented or unreplicated trials [85,96]. Across studies, a clear pattern emerges in which structured and replicated designs produced moderate to low experimental CV values, while unreplicated or augmented designs show greater variability with mostly moderate to high values.

#### 4.1.3. Broad-Sense Heritability

Heritability estimates, reported in a subset of studies, further characterized the genetic contribution to the observed variation. Estimates varied across experimental conditions, population structure, replication, and analytical approaches. Studies conducted under single environments using designs such as RCBD and lattice with adequate replication (2–3 replications) generally reported moderate (>30–60%) to high (>60%) heritability for most agro-morphological traits, particularly plant height, flowering traits, and kernel-related traits [67,83,94,103,104], whereas studies using augmented designs reported moderate heritability estimates [107]. Traits such as grain yield and some yield components frequently exhibited moderate to low (<30%) heritability, particularly in multi-environment trials [64,68,101,105]. Studies conducted across multiple environments, seasons, or stress conditions reported more variable heritability estimates [68,89]. In addition, differences in analytical approaches, including ANOVA-based models and linear mixed models (REML/BLUP), were also observed [67,83,101,103,105], thus limiting the direct comparability of heritability estimates across studies.

### 4.2. Multivariate Structure of Phenotypic Diversity

#### 4.2.1. Trait Associations: Correlation Analysis

Across studies, multivariate analyses consistently showed that phenotypic variation among maize landraces is structured primarily around flowering traits, plant architecture, and yield components. Correlation analyses revealed strong positive associations between plant height and ear height (r > 0.8) and between anthesis and silking [95,96,107,109,113]. Grain yield was frequently positively correlated with ear length, kernel number, and grain weight, while negative associations were commonly observed with anthesis-silking interval and delayed flowering [88,89,103,105,113]. These correlation patterns reinforce the interconnected roles of flowering, plant architecture, and ear and kernel traits in shaping yield expression [65,95,111]. These patterns support the selection of high-yielding genotypes together with yield-enhancing traits. Thus, supporting the accumulation of favourable traits in breeding populations.

#### 4.2.2. Principal Component Analysis (PCA)

PCA was widely used to summarize the major sources of phenotypic variation among maize landraces. Across studies, a minimum of two and a maximum of seven principal components were reported, explaining approximately 55–90% of the total variation [67,76,85,92,96,102]. In most cases, the first two principal components explained the largest share of variation, while the first four components together accounted for most of the total variability. Variation in the distribution of explained variance across components was associated with the number of traits evaluated, with studies assessing larger trait sets showing a broader spread of variation across multiple components [84,85,91], whereas studies with fewer traits showed greater concentration of variance in the first two components [76,92,99,104]. Across studies, PC1 was most frequently associated with phenological and plant architecture traits, including flowering time, plant height, ear height, vegetative growth, and yield-related attributes [88,93,97,98]. PC2 commonly represented ear and kernel characteristics such as ear length, ear diameter, kernel row number, kernel size, kernel weight, and grain number per row [76,82,85,94,95,96,107]. In some studies, PC2 was instead associated with flowering or tassel-related traits, including flowering duration, tassel branching, and tassel size [73,78,102,107,109], reflecting differences in trait sets and biological emphasis across experiments. Across studies, a consistent pattern was observed in which phenological traits and plant architecture dominate the primary axes of variation, while ear and kernel traits contribute to secondary components.

#### 4.2.3. Cluster Analysis and Population Grouping

Cluster analysis further clarified the phenotypic structure of maize landrace diversity by grouping genotypes with similar trait profiles. Across studies, landraces were typically classified into two to nine clusters, although two to four clusters were most commonly reported [67,76,80,88,92,95,96,108,113]. Studies identifying a larger number of clusters generally evaluated larger or more diverse germplasm panels or broader trait sets, resulting in five to seven clusters in several cases [64,82,85,86,90,102,107] and up to eight or nine clusters in a few studies [107,112]. Cluster differentiation was most frequently driven by combinations of flowering time, plant architecture, and yield-related traits, including plant height, ear height, ear characteristics, and kernel attributes [67,78,92,93,96,108,113]. In some studies, clustering patterns were also associated with geographic origin, racial classification, or ecological adaptation of the landraces, indicating that phenotypic diversity reflects both genetic variability and environmental influences [71,80,100,102,110,112]. These cluster groupings across studies can be used to guide the selection of diverse germplasm for specific breeding objectives and improvement programs.

Together, correlation, PCA, and cluster analyses demonstrate that maize landrace diversity is structured around key interconnected traits related to flowering, plant architecture, and yield, which collectively determine performance and adaptation. The convergence of results across these multivariate approaches highlights consistent biological patterns despite differences in trait sets, study size, and experimental focus. However, variation among studies in experimental design, number of evaluation environments, replication levels, germplasm composition, and statistical models can influence the magnitude of reported coefficients of variation, heritability estimates, PCA variance explained, and correlation coefficients. Consequently, these statistical measures should be interpreted as indicators of general patterns of phenotypic diversity rather than strictly comparable numerical values across studies, while still providing a basis for identifying key traits and guiding selection and germplasm improvement in maize breeding programs.

### 4.3. Heterogeneity and Sources of Variation

Considerable methodological heterogeneity was observed across studies, driven primarily by differences in environments, numbers of genotypes, and traits assessed. Multi-location or multi-season studies generally reported broader phenotypic variation than single-site trials, reflecting increased environmental sampling. Variation in replication levels, experimental designs, and descriptor systems further contributed to heterogeneity and influenced the precision and comparability of phenotypic diversity estimates.

Despite this heterogeneity, consistent trait patterns and statistical outcomes were reported across most studies. In particular, studies combining phenotypic and molecular data often showed clearer differentiation among landraces, supporting the consistency of observed diversity patterns and highlighting the complementary value of integrative approaches.

### 4.4. Sensitivity of Findings

The main synthesis outcomes were not sensitive to individual studies or specific experimental designs. Key patterns, including significant genotypic differences, high variability in flowering, plant architecture, and yield-related traits, and consistent trait associations, were observed across studies varying widely in sample size, number of traits, and experimental layout. Studies with smaller panels or fewer traits generally reported reduced resolution but did not contradict overall trends. Similarly, exclusion of studies lacking replication or using simplified designs did not alter the direction of the main findings, indicating that the main patterns were consistent across methodological contexts.

### 4.5. Reporting Bias and Certainty of Evidence

Some degree of reporting inconsistency was observed across the reviewed studies, particularly in the depth and completeness of methodological and statistical information provided. While most studies reported ANOVA outcomes and descriptive measures of variability, information on model assumptions and diagnostic checks was rarely documented. In addition, non-significant results were often reported with less emphasis than significant findings. Nevertheless, the inclusion of diverse study designs, environments, and trait sets, together with the consistency of phenotypic patterns observed across independent studies, suggests that these reporting limitations are unlikely to substantially affect the overall conclusions of the review.

Overall, the certainty of evidence was assessed as moderate for conclusions related to phenotypic variability, trait correlations, and landrace differentiation based on agro-morphological traits. This assessment is supported by the large number of independent field-based studies, consistent findings across diverse environments, and repeated use of standardized descriptor systems. Certainty was lower for yield-related stability and genotype × environment interaction effects due to environmental sensitivity and inconsistent reporting across studies. Nevertheless, the convergence of results across multiple analytical approaches strengthens confidence in the main conclusions of this review.

## 5. Discussion

### 5.1. Patterns of Phenotypic Diversity

The synthesized evidence indicates that phenotypic diversity in maize landraces is extensive and structured, consistent with patterns of adaptation and selection reported in previous studies [32,34,114]. The recurrent importance of flowering time, plant architecture, and ear and kernel traits across diverse environments suggests that these traits represent key adaptive axes through which landraces respond to agro-ecological conditions [88,112,115,116]. Their dominance in multivariate analyses further indicates that adaptation to season length, stress exposure, and management practices plays an important role in shaping landrace differentiation [92,93,107,108,113]. Traits such as earliness, plant stature, and ear placement have been reported as targets of farmer selection in low-input systems, where synchronization with rainfall and resilience to abiotic stress are critical [117,118]. Furthermore, the observed clustering of landraces by maturity class, yield potential, and, in some cases, geographic or racial origin further supports the role of local adaptation and seed exchange networks in structuring diversity [71,80,100,102,110]. However, rather than forming discrete genetic units, many landrace populations appear to occupy overlapping phenotypic space, reflecting continuous selection and gene flow among farmer-managed populations [66,119,120]. Therefore, maize landraces represent dynamic populations influenced by both genetic variability and environmental conditions, with significant potential for breeding under diverse environments.

### 5.2. Methodological Heterogeneity and Reporting Inconsistencies

Despite consistent evidence of phenotypic diversity among maize landraces, methodological heterogeneity across studies limits the comparability and reproducibility of diversity estimates. Differences in experimental design, replication, and statistical analysis directly influence the precision and interpretation of results. Univariate analyses based on ANOVA consistently detected significant variation; however, studies using replicated designs such as RCBD and lattice designs provided more reliable estimates by enabling clearer partitioning of genetic, environmental, and genotype × environment effects [80,82,83,108]. In contrast, augmented and unreplicated designs provide no replication for test entries [84,99,107], which may influence variation in the precision and interpretability of diversity estimates. Significant differences were observed for most agro-morphological traits, confirming substantial phenotypic differentiation within landrace collections. Non-significant results were also reported for some traits, but these were likely associated with environmental effects or genotype × environment interactions rather than a lack of diversity. Variability was consistently detected across studies, although its precise estimation depended on the experimental design employed.

This pattern was further reflected in the reporting of CV. Experimental CV derived from ANOVA served as an indicator of trial precision and was generally lower and more stable in replicated designs [88,100,104], whereas higher and more variable CV values in unreplicated trials reflected reduced precision due to limited replication [77,84,93]. Importantly, differences in how CV was calculated and reported (experimental vs. descriptive) limited direct comparability across studies, as several studies reported CV as a measure of phenotypic variability derived from descriptive statistics (standard deviation/mean), where CV reflected overall variability among genotypes rather than experimental precision. Considering trait classification, phenological traits generally exhibited lower CV values [80,108,110], suggesting more stable expression, while traits such as anthesis-silking interval and grain yield [74,87,88] often showed higher variability due to stronger environmental influence.

Similarly, heritability estimates varied widely depending on experimental conditions, number of environments, and analytical approaches. Studies conducted under single environments with adequate replication often reported moderate to high heritability for traits such as flowering time, plant height, and kernel characteristics [67,83,94], whereas multi-environment trials tended to report lower heritability for complex traits such as grain yield, partly due to genotype × environment interaction [64,68,105]. Differences in statistical methods also influenced estimates, as ANOVA-based approaches may be more sensitive to experimental error and unbalanced data [66,107], whereas mixed models (REML/BLUP) can account for complex variance structures and provide more stable estimates by adjusting for environmental effects and genotype × environment interaction [89,101], which may limit direct comparability across studies. These findings indicate that heritability estimates in maize landraces are influenced not only by the underlying genetic variability of the population but also by experimental design, replication, and environmental conditions, highlighting the importance of structured experimental designs with adequate replication for obtaining reliable estimates for breeding and selection purposes.

Trait assessment also lacked uniformity. Although many studies evaluated similar agro-morphological traits, descriptor systems were frequently not reported, with 19 studies failing to specify the trait descriptors used. Even among studies referencing CIMMYT/IBPGR guidelines, inconsistent trait nomenclature was common. For example, equivalent traits were reported under different names (e.g., “male flowering,” “anthesis,” “anthesis date,” “days to 50% anthesis” or “days to tasseling”; “ear height” versus “uppermost ear height”; “floral asynchrony” versus “ASI”; and “100 KW” versus “hundred kernel weight”), complicating cross-study synthesis and requiring extensive standardization during data extraction [64,69,75,82,86,91,97,100,102,108,109,111].

Furthermore, incomplete reporting of environmental metadata further constrained interpretation. Specifically, key variables such as rainfall, temperature, soil type, plot dimensions, plant density, and sampling intensity were frequently missing or inconsistently documented, limiting reproducibility and reducing the ability to interpret environmental effects across studies [97,106,121,122]. Similarly, only a minority of studies (13) reported heritability estimates, and few explicitly quantified genotype × environment interactions, despite their central role in evaluating trait stability and breeding value [71,72,75,123].

Finally, incomplete bibliographic reporting, including missing Digital Object Identifiers (DOIs), reduced traceability and hindered verification of some sources. Collectively, these methodological and reporting gaps do not undermine the evidence for phenotypic diversity in maize landraces, but they substantially limit cross-regional comparison, quantitative synthesis, and the effective translation of diversity estimates into breeding and conservation applications, highlighting the need for greater standardization and reporting.

### 5.3. Implications for Breeding and Conservation

The traits most frequently identified as major contributors to phenotypic variation, particularly flowering time, plant architecture, and yield components, represent key targets for selection in breeding programs because they combine relatively stable expression with significant contributions to overall performance. Flowering traits, namely days to anthesis, days to silking, and the anthesis-silking interval, are important for developing varieties adapted to local growing seasons and for improving drought escape and stress tolerance [89,124]. Plant architecture traits, including plant height and ear height, influence lodging resistance and suitability for different management systems, making them important selection criteria [125]. Ear and kernel traits, such as ear length, kernel number, and kernel weight, are directly associated with yield potential and are commonly used to identify high-performing genotypes [79,103]. Although grain yield remains the primary target trait, its strong environmental sensitivity often requires indirect selection through more stable yield-related traits. Therefore, the combined evaluation of these agro-morphological traits supports the identification of promising landraces, the selection of parents for crossing, and the improvement of adaptation and productivity in maize breeding programs. In addition, the use of rigorous experimental designs, appropriate statistical models, and multi-environment testing is essential for generating reliable diversity estimates that can effectively support germplasm utilization.

From a conservation perspective, the erosion of traditional varieties and seed exchange systems threatens this diversity, emphasizing the need for integrated ex situ and in situ conservation strategies, while strengthening community seed systems and participatory breeding approaches will be essential for maintaining functionally important diversity [126,127]. The identified methodological differences further highlight the need for harmonized phenotyping protocols, standardized descriptors, and improved data transparency to enhance the use of landrace diversity in both breeding and conservation.

### 5.4. Limitations of the Review Process

Although this review provides broad global coverage of phenotypic diversity studies in maize landraces, several limitations should be acknowledged. First, restricting the search to English-language publications may have resulted in the exclusion of relevant studies published in other languages, particularly locally disseminated research from regions with rich maize landrace diversity. This restriction may introduce language bias and potentially favor studies published in English-speaking contexts. To explore the possible magnitude of this effect, exploratory searches were conducted across the databases used in this review. Searches in ScienceDirect, PubMed, and AGRIS retrieved relatively few non-English records (for instance, one Spanish and two French records in ScienceDirect, none in PubMed, and 12 Spanish and 8 Portuguese records in AGRIS). In contrast, exploratory searches conducted in Google Scholar using translated search terms in Spanish, Portuguese, and Chinese returned substantially larger numbers of results (approximately 4120, 2210, and 1960 records, respectively). This difference likely reflects the broader indexing coverage of Google Scholar, which includes theses, institutional repositories, conference papers, and other grey literature, as well as the absence of an explicit language filter. However, these figures represent preliminary search results prior to screening and may include duplicates or studies that would not meet the eligibility criteria. Therefore, they provide only an approximate indication that some potentially relevant literature may exist in languages other than English that were not captured within the scope of this review.

Secondly, limited access to subscription-based databases such as Scopus, Web of Science, and CABI may have reduced the completeness of retrieved records, and some studies published in regional or non-indexed journals may not have been captured. Despite these limitations, the consistency of phenotypic patterns observed across 50 studies from 30 countries supports the robustness of the major conclusions drawn in this review. Furthermore, the transparent and systematic application of PRISMA-based screening, standardized data extraction, and explicit reporting of methodological gaps provides a reliable synthesis of current research trends.

## 6. Conclusions

This systematic review demonstrates that maize landraces exhibit extensive phenotypic diversity based on agro-morphological traits across different regions and study locations. Owing to the high heterogeneity of maize landraces, environmental factors strongly influence phenotypic expression, which in turn affects selection decisions. The reviewed studies show that structured experimental designs help reduce environmental effects and improve the reliability of phenotypic evaluation for breeding. The frequent occurrence of moderate to high heritability for several morphological traits suggests a genetic contribution to their expression, although phenotypic variation alone does not fully capture underlying genetic diversity. These traits reflect the combined influence of genetic factors, environmental conditions, and genotype × environment interactions, highlighting the complexity of genotype-phenotype relationships. Additionally, the greater environmental sensitivity of complex traits such as grain yield emphasizes the need for multi-environment evaluation to identify stable and broadly adapted genotypes.

Beyond synthesizing diversity patterns, this review also identifies important methodological and reporting gaps that limit cross-study comparability and hinder the translation of phenotypic evidence into breeding and conservation practice. Improved standardization of phenotyping and environmental data will enhance reproducibility and interpretation of diversity assessments. Importantly, integrating phenotypic data with molecular and genomic information is necessary to validate and better interpret the genetic basis of observed variation and improve the identification of useful traits for breeding. Future studies should embrace the integration of high-resolution genomic data with standardized phenotypic evaluations, which will improve the characterization of landrace diversity, enhance identification of adaptive genetic variation, and support more efficient selection in breeding programs.

## 7. Recommendations

The methodological inconsistencies identified in this review highlight the need for a harmonized approach to agro-morphological diversity assessment. Linking experimental design, phenotyping, environmental characterization, and genetic analysis can improve comparability and reproducibility across studies. The following recommendations are organized into four interconnected components.

(1) Experimental design and population structuring.

Phenotypic evaluation should be conducted using replicated and spatially adjusted designs such as, randomized complete block and alpha-lattice, which ensure proper replication of entries in order to control environmental variation and errors. The use of augmented design in the situation of evaluating a large population and limited resources should be incorporated with checks of different phenotypic performance so as to evaluate environmental variation through replication of the checks. Adequate replication and appropriate use of checks are essential to control environmental variation and ensure reliable estimation of genetic effects. In addition, a clear definition of the study population, including landrace classification and the role of checks varieties, is necessary to support consistent interpretation of diversity patterns.

(2) Standardized phenotypic characterization.

To address inconsistencies in trait definitions, studies should adopt internationally recognized descriptor systems, particularly CIMMYT/IBPGR descriptors, and apply standardized trait nomenclature. Core trait sets should prioritize phenological, architectural, and yield-related traits identified in this review as key drivers of diversity. Consistent phenotyping protocols will enhance cross-study comparability and enable meaningful synthesis of agro-morphological data.

(3) Environmental and metadata integration.

Given the strong influence of environmental conditions on trait expression, comprehensive metadata should be systematically recorded, including climatic variables, soil characteristics, management practices, and sampling strategies. Multi-environment evaluation should be prioritized to capture genotype × environment interactions and improve the assessment of trait stability. Standardized metadata reporting will facilitate interpretation of phenotypic variation and enable more robust cross-location comparisons.

(4) Integrated statistical and genomic analysis.

To improve genetic interpretability, phenotypic data should be analyzed using a combination of univariate (ANOVA) and multivariate approaches (PCA, clustering), together with estimation of genetic parameters such as heritability and coefficients of variation to explain population diversity and trait relationships. In addition, incorporating genomic tools such as marker-assisted selection (MAS) and genome-wide association studies (GWAS) with phenotypic data will facilitate understanding the underlying genetic variation at the molecular level. This integration enables the identification and selection of genotypes using marker-trait associations and supports genotype-phenotype inference for key adaptive traits. Use of this integrative approach would enhance the reliability and comparability of diversity estimates and improve the practical use of maize landrace for breeding and conservation programs.

## Figures and Tables

**Table 1:** Study-level characteristics and key findings of maize landrace phenotypic diversity studies included in the review, arranged chronologically by year of publication.

Study	Year	Number of Genotypes	Design	Number of Traits	Statistical Analysis	Key Findings
[64]	2001	100	RCBD	22	ANOVA, Heritability, PCA, Cluster	Significant differences; high heritability for cob weight, kernel rows; PCA-based clustering into 7 groups
[65]	2002	32	RCBD	25	ANOVA, Pearson correlation	Significant differences among races and accessions; strong trait correlations
[66]	2004	31	No standard design (two-replicate hierarchical design)	15	ANOVA, Genetic variability (GCV/PCV), Cluster	Significant population differentiation; moderate-high variability
[67]	2005	180	RCBD	15	ANOVA, Heritability, PCA, Cluster	Significant variation; high heritability but low for yield traits; 3 key PCs (70%); 4 clusters
[68]	2007	85	Split–split plot	9	ANOVA, Pearson correlation, Heritability	Significant differences; strong correlations; moderate heritability
[69]	2008	124	RCBD	20	ANOVA, Cluster	Variation confined to a few traits; 5 geographic clusters
[70]	2008	43	RCBD	41	Descriptive, PCA, Cluster	2 PCs (31.8%); 4 groups; white types taller & higher yielding;
[71]	2008	54	RCBD	20	ANOVA, PCA, Cluster	Significant differences; 5 PCs; 4 clusters by ear type/origin
[72]	2009	40	No standard design (randomized grouping)	18	ANOVA, PCA, Cluster	Significant differences; 4 key PCs-earliness, ear shape, yield, seed size; 4 clusters
[73]	2009	21	RCBD	15	Descriptive, ANOVA, PCA, Cluster	Highest CVs in tassel/kernel traits; significant variation; 5 PCs (83.7%); 2 clusters
[74]	2009	57	RCBD	7	ANOVA	Significant differences among accessions for yield traits
[75]	2009	104	RCBD	12	ANOVA, PCA, Cluster	Significant differences; PCA separated landraces by yield and morphology; 2–3 clusters
[76]	2010	48	Alpha Lattice	9	ANOVA, PCA, Cluster	Significant differences; 2 key PCs; 4 yield- based clusters
[77]	2010	10	No standard design (single-row plots, no replication across blocks)	20	ANOVA, Descriptive	Significant differences in height, leaf, ear, and kernel traits
[78]	2012	77	Simple lattice design	28	ANOVA, PCA, Cluster	Significant differences; 4 key PCs (69.8%); groups by yield, flowering, kernels
[79]	2012	18	No standard design (Characterization field experiment)	25	Descriptive, Pearson correlation, Cluster	Strong trait correlations; clear divergent clusters
[80]	2013	30	RCBD	7	ANOVA, PCA, Cluster	Significant differences; flowering & leaves key traits; 3 clusters
[81]	2014	21	RCBD	18	Descriptive, ANOVA, PCA, Cluster	High variability; 4 PCs (80.9%); weak separation by agro-ecological origin
[82]	2014	100	Simple Lattice design	40	ANOVA, PCA, Cluster, Pearson correlation	Significant differences; 3 key PCs; 7 distinct groups
[83]	2015	9	RCBD	7	ANOVA, Heritability & Genetic variability (GCV/PCV); Cluster	Significant differences; high heritability (>79%); 3 clusters
[84]	2015	51	Augmented Block Design	16	ANOVA, PCA, Cluster	Significant differences; 6 PCs; 4 geography-based trait clusters
[85]	2015	153	Augmented Block Design	34	Descriptive, Pearson correlation, PCA, Cluster	High variability; strong flowering correlations; flowering drives PC1; 5 clusters
[86]	2015	118	Simple Lattice design	22	ANOVA, Descriptive, PCA, Cluster	Significant differences; wide variability; 3 key PCs; 5 yield-based groups
[87]	2015	76	Simple Lattice design	7	ANOVA, Cluster	Significant differences; regional grouping
[88]	2016	35	RCBD	27	ANOVA, Pearson correlation, PCA, Cluster	Significant differences; yield linked to ear leaf width & kernel traits; 4 key PCs (86.7%); 4 clusters
[89]	2016	78	Alpha Lattice	13	Pearson correlation, Genetic variability (GCV/PCV)	High genetic variability; drought reduced yield; strong kernel-yield correlation
[90]	2017	91	incomplete randomized block	16	ANOVA, Cluster	Significant differences; 5 groups-locals distinct from improved types
[91]	2017	50	RCBD	31	ANOVA, PCA, Cluster	Significant differences; 3 key PCs separate yield/leaf, flowering/ASI, cob and kernel traits; 4 distinct morphological clusters
[92]	2017	60	RCBD	26	ANOVA, PCA, Cluster	Significant differences; PCs driven by architecture and kernel traits; 2 clusters by maturity/yield
[93]	2017	75	Augmented Block Design	12	ANOVA, PCA, Cluster	Significant differences; 3 key PCs; 5 morphological clusters
[94]	2017	48	RCBD	15	PCA, Cluster, Pearson correlation & Heritability	4 key PCs driven by yield, height, and micronutrients; High heritability; 2 clusters
[95]	2017	34	RCBD	41	Descriptive, Pearson correlation, PCA, Cluster	Wide trait variability; 3 key PCs (73%); 4 clusters
[96]	2018	47	Augmented Block Design	24	ANOVA, Pearson correlation, PCA, Cluster	Significant differences; strong correlations; 3 key PCs (65%); 3 clusters
[97]	2018	50	No standard design (evaluated in experimental plots, glasshouse + field; replicates)	16	PCA, Cluster	4 key PCs (86%) ear/cob weight & diameters; 3 clusters based on ear traits
[98]	2018	28	RCBD	21	PCA, Cluster	5 key PCs (80%); 2 clusters separated by height and flowering
[99]	2019	56	Augmented Block Design	14	ANOVA, PCA	Significant differences across locations; 2 key PCs driven by flowering, height, ear, and yield
[100]	2019	144	Simple Lattice design	33	ANOVA, Pearson correlation, Cluster	Significant differences; key traits selected; 6 clusters
[101]	2019	36	Alpha Lattice	16	ANOVA, Heritability; Pearson correlation; Cluster,	Significant differences; moderate-high heritability; strong yield correlations; stress-based clusters
[102]	2020	196	Alpha Lattice	26	ANOVA, PCA, Cluster	Significant variation; 3 key PCs (76.5%); 5 clusters by maturity/origin
[103]	2020	13	RCBD	10	ANOVA, Heritability, Pearson correlation, PCA, Cluster	Significant differences; high heritability; strong correlations; PCs (88.1%) driven by kernel traits; 3 clusters
[104]	2021	59	Alpha lattice	13	ANOVA, Heritability, Pearson correlation, PCA, Cluster	Significant differences; moderate heritability; 2 PCs; 2 clusters
[105]	2021	39	RCBD	12	ANOVA, Heritability, Pearson correlation, PCA, Cluster	Significant differences; high heritability for weight & diameter; 5 PCs; 7 clusters;
[106]	2021	298	RCBD	26	Cluster	5 clusters; moderate redundancy in both groups
[107]	2021	99	Augmented Block Design	30	ANOVA, heritability, Pearson correlation, PCA, Cluster	Significant differences; high heritability, strong flowering and height correlations; 8 clusters
[108]	2022	70	Alpha Lattice	18	Descriptive, ANOVA, PCA, Cluster,	High trait variability; significant differences; 3 key PCs (78.7%); 2 clusters
[109]	2022	30	RCBD	19	Descriptive, Pearson correlation, PCA, Cluster	High variability; strong correlations; 3 key PCs (58.6%); 2 clusters
[110]	2023	64	Simple Lattice design	24	ANOVA, Pearson correlation, PCA, Cluster	Significant differences; 4 clusters by grain color/earliness
[111]	2024	50	No standard design (single-site validation field trial)	35	Pearson correlation, PCA	Strong correlations among flowering time, plant architecture, and ear traits; 2 key PCs (65.41%)
[112]	2024	588	No standard design	6	ANOVA, PCA, Cluster	Significant differences; groups align with geography/genetics
[113]	2025	36	RCBD	15	PCA, Pearson correlation, Cluster	4 clusters; 4 PCs (75.2%) earliness & ear shape structure diversity

RCBD = randomized complete block design; ANOVA = analysis of variance; PCA = principal component analysis; PCs = principal components; GCV = genotypic coefficient of variation; PCV = phenotypic coefficient of variation; ASI = anthesis-silking interval.

**Table 2:** Aggregated characteristics of studies on phenotypic diversity of maize landraces.

Characteristic	Summary
Type of study	Experimental (49), Experimental + secondary data (1)
Trial type	Field trial (34), Field + molecular (15), field trial + historical data (1)
Genotypes assessed	9–588 (median 53)
Traits evaluated	6–41 (average 19)
Replications	2 reps (17), 3 reps (20)
Checks included	27 (improved varieties hybrid or landraces); 23 (not included)
Plot setup and sampling	Plot length 3 m (5), 5 m (11), 6 m (7); Rows per plot 1 row (18), 2 rows (11); 1–4 rows most common; sample size 10 plants (19), 5 plants (11)
Descriptors used	CIMMYT/IBPGR (27), Bioversity International (2011) (2), ICAR-NBPGR minimal (2000) (1), NBPGR minimal descriptors (1), not reported (19)
Spacing and planting density	0.75 × 0.20–0.40 m; planting density 44,000–53,000 plants/ha
Agro-ecological coverage	Tropical, subtropical, and temperate zones across four continents

CIMMYT–IBPGR = International Maize and Wheat Improvement Center—International Board for Plant Genetic Resources; NBPGR = National Bureau of Plant Genetic Resources.

**Table 3:** Frequency of experimental designs used in studies on phenotypic diversity of maize landraces.

Experimental Design	Frequency	Percentage
RCBD	23	46.00%
Simple Lattice	6	12.00%
Alpha Lattice	6	12.00%
Augmented	6	12.00%
Others	9	18.00%
Total	50	100%

## Data Availability

All data supporting this systematic review are included in the Supplementary Materials and available in the Open Science Framework (OSF) repository under https://doi.org/10.17605/OSF.IO/YHBN7.

## Supplementary Materials

The following supporting information can be downloaded at: https://www.mdpi.com/article/10.3390/genes17040413/s1, Figure S1: presents the risk-of-bias traffic light plots for all 50 included studies; Table S1: provides the full evidence table of studies reporting phenotypic diversity of maize landraces from 2000 to 2025 and the key findings; Table S2: contains additional study characteristics, including number of trial sites, number of seasons, elevation and climatic conditions; Table S3: summarizes the geographic distribution of studies by region.

## Abbreviations

The following abbreviations are used in this manuscript:

AGRIS	International System for Agricultural Science and Technology
ANOVA	Analysis of Variance
CIMMYT–IBPGR	International Maize and Wheat Improvement Center—International Board for Plant Genetic Resources
NBPGR	National Bureau of Plant Genetic Resources
PCA	Principal Component Analysis
PICOTS	Population, Intervention, Comparator, Outcomes, Timing, and Setting
PRISMA	Preferred Reporting Items for Systematic Reviews and Meta-Analyses
PubMed	Public/Publisher MEDLINE Database
RCBD	Randomized Complete Block Design
RoBVis	Risk-of-Bias Visualization Tool
