# Supplementary material for: Phenotypic Diversity in Maize Landraces: A Systematic Review of Global Patterns, Methodological Approaches, and Implications for Breeding
Source: Genes (Basel). 2026 Mar 31;17(4):413. doi: 10.3390/genes17040413 (PMC13115568; doi:10.3390/genes17040413)
Supplement: Supplementary file 1 [file genes-17-00413-s001.zip › Table S1. provides the full evidence table of studies reporting phenotypic diversity of maize landraces from 2000 to 2025 and the key findings.docx]

**Table S1:** Comprehensive evidence table summarizing studies on phenotypic diversity of maize landraces and their key findings. Author names are repeated according to the number of analysis methods reported.

| **Study** | **Type of Trial** | **Number of genotypes** | **Design** | **Descriptor Used** | **Number of Traits** | **Statistical Analysis** | **Key Findings** |
| --- | --- | --- | --- | --- | --- | --- | --- |
| N’da et al., 2022 | Field trial | 70 | Alpha Lattice | CIMMYT/IBPGR (1991) | 18 | ANOVA | Significant differences (p < 0.001) among genotypes. |
| N’da et al., 2022 | Field trial | 70 | Alpha Lattice | CIMMYT/IBPGR (1991) | 18 | Cluster Analysis | 2 groups: late/tall/high-yield vs early/short/medium-yield |
| N’da et al., 2022 | Field trial | 70 | Alpha Lattice | CIMMYT/IBPGR (1991) | 18 | Descriptive Statistics | High trait variability (CV ≈ 55% for tertiary branching) |
| N’da et al., 2022 | Field trial | 70 | Alpha Lattice | CIMMYT/IBPGR (1991) | 18 | PCA | 3 PCs explained 78.7% variance – phenology, yield, growth traits |
| Nelimor et al., 2020 | Field trial | 196 | Alpha Lattice | CIMMYT/IBPGR (1991) | 26 | ANOVA | Significant differences (p < 0.001) across 26 traits; high repeatability (0.57–0.99) |
| Nelimor et al., 2020 | Field trial | 196 | Alpha Lattice | CIMMYT/IBPGR (1991) | 26 | Cluster Analysis | 5 clusters formed mainly by maturity and origin (Sahel vs Coastal) |
| Nelimor et al., 2020 | Field trial | 196 | Alpha Lattice | CIMMYT/IBPGR (1991) | 26 | PCA | 3 PCs explained 76.5% variance – phenology/yield, flowering/stay-green, tassel/kernel traits |
| Aci et al., 2018 | Field trial | 47 | Augmented Block Design | CIMMYT/IBPGR (1991) | 24 | ANOVA | Significant differences (p < 0.05) for 13 traits including flowering, height, and yield traits |
| Aci et al., 2018 | Field trial | 47 | Augmented Block Design | CIMMYT/IBPGR (1991) | 24 | Cluster Analysis | 3 clusters: (1) early/short/low-yield, (2) large-kernel/high 1000-grain wt, (3) vigorous/high-yield |
| Aci et al., 2018 | Field trial | 47 | Augmented Block Design | CIMMYT/IBPGR (1991) | 24 | Descriptive statistics | Wide variability in ASI (125%), ear height (44%), and yield (36%); kernel traits less variable |
| Aci et al., 2018 | Field trial | 47 | Augmented Block Design | CIMMYT/IBPGR (1991) | 24 | PCA | 3 PCs explained 65% variance – PC1 (44%) ear/height/yield, PC2 (11%) kernel size, PC3 (9%) vigor/ASI |
| Aci et al., 2018 | Field trial | 47 | Augmented Block Design | CIMMYT/IBPGR (1991) | 24 | Pearson Correlation Analysis | Strong positive r = 0.97 (silking–anthesis) and r = 0.92 (plant–ear height); yield linked to height and flowering |
| Beyene et al., 2005 | Field trial | 180 | RCBD | CIMMYT/IBPGR (1991) | 15 | ANOVA | Significant differences (p < 0.05–0.01) across 15 traits, confirming genetic diversity |
| Beyene et al., 2005 | Field trial | 180 | RCBD | CIMMYT/IBPGR (1991) | 15 | PCA | 3 PCs explained ~70% variance – PC1 flowering/size, PC2 yield components, PC3 vegetative traits |
| Beyene et al., 2005 | Field trial | 180 | RCBD | CIMMYT/IBPGR (1991) | 15 | Variability Estimates | High GCV and heritability for leaf number, maturity, and height; yield traits had low heritability |
| Beyene et al., 2013 | Field trial | 180 | RCBD | CIMMYT/IBPGR (1991) | 15 | Cluster Analysis | 4 clusters: early/short/high-yield vs tall/late/low-yield groups, showing clear divergence |
| Obeng-Antwi et al., 2012 | Field trial | 77 | Simple lattice design | not reported | 28 | ANOVA | Significant differences (p < 0.01) for all traits across accessions |
| Obeng-Antwi et al., 2012 | Field trial | 77 | Simple lattice design | not reported | 28 | Cluster Analysis | Clusters formed using 13 traits; groups differentiated by yield potential, flowering time, plant height, and kernel traits |
| Obeng-Antwi et al., 2012 | Field trial | 77 | Simple lattice design | not reported | 28 | PCA | 4 PCs explained 69.8% variance – PC1 yield/ear traits, PC2 flowering/vegetative, PC3–PC4 ear/kernel traits |
| Stephen et al., 2016 | Field trial | 35 | RCBD | CIMMYT/IBPGR (1991) | 27 | ANOVA | Significant differences within and between populations; mid-altitude group most variable; yield 0.7–12.5 Mg ha⁻¹ |
| Stephen et al., 2016 | Field trial | 35 | RCBD | CIMMYT/IBPGR (1991) | 27 | Cluster Analysis | 4 clusters by geographic origin; Cluster II tall, large-tasseled, highest yield (~5.2 Mg ha⁻¹) |
| Stephen et al., 2016 | Field trial | 35 | RCBD | CIMMYT/IBPGR (1991) | 27 | PCA | 4 PCs explained 86.7% variance – PC1 (40%) plant architecture/stay-green/yield; PC3 inverse maturity-yield relationship |
| Stephen et al., 2016 | Field trial | 35 | RCBD | CIMMYT/IBPGR (1991) | 27 | Pearson Correlation Analysis | Yield positively correlated with ear leaf width, plant height, and kernel traits; negatively with ASI |
| Ndiso et al., 2013 | Field trial | 30 | RCBD | not reported | 7 | ANOVA | Significant differences for leaves and flowering traits; ears per plant and yield not significant |
| Ndiso et al., 2013 | Field trial | 30 | RCBD | not reported | 7 | Cluster Analysis | 2 main clusters (coastal landraces vs checks) with sub-clusters by geographic origin; diversity linked to location |
| Ndiso et al., 2013 | Field trial | 30 | RCBD | not reported | 7 | PCA | no explicit trait loadings reported, Broad diversity observed with flowering and leaf traits likely influenced grouping |
| Hafiz et al., 2017 | Field trial | 91 | incomplete randomized block | CIMMYT/IBPGR (1991) | 16 | ANOVA | Significant differences (p < 0.001) for all traits; highest variability in plant height, ear height, and germination time |
| Hafiz et al., 2017 | Field trial | 91 | incomplete randomized block | CIMMYT/IBPGR (1991) | 16 | Cluster Analysis | 5 groups identified; local accessions dominated clusters G1, G2, and G4, distinct from improved types |
| Kabululu et al., 2017 | Field trial | 50 | RCBD | CIMMYT/IBPGR (1991) | 31 | ANOVA | Significant differences (p < 0.05) across all traits, confirming morphological diversity |
| Kabululu et al., 2017 | Field trial | 50 | RCBD | CIMMYT/IBPGR (1991) | 31 | Cluster Analysis | 4 clusters: (I) tall/early/high-yield, (II) unique CML 442, (III) tall/long ASI, (IV) short/late/low-yield |
| Kabululu et al., 2017 | Field trial | 50 | RCBD | CIMMYT/IBPGR (1991) | 31 | PCA | 6 PCs explained 59.8% variance – PC1 yield/leaf/ear traits, PC2 flowering/height/ASI, PC3 cob and kernel traits |
| Twumasi et al., 2017 | Field trial | 60 | RCBD | CIMMYT/IBPGR (1991) | 26 | ANOVA | Significantdifferences (p < 0.01) for most quantitative traits |
| Twumasi et al., 2017 | Field trial | 60 | RCBD | CIMMYT/IBPGR (1991) | 26 | Cluster Analysis | 2 clusters differentiated by maturity, architecture, kernel weight, and yield |
| Twumasi et al., 2017 | Field trial | 60 | RCBD | CIMMYT/IBPGR (1991) | 26 | PCA | 2 PCs explained 67.9% variance – PC1 earliness/architecture, PC2 yield/kernel traits |
| Defacio et al., 2025 | Field trial and molecular characterization | 36 | RCBD | CIMMYT/IBPGR (1991) | 15 | Cluster Analysis | 4 clusters: (1) high-yield OPVs, (2) low-yield, (3) average/short, (4) tall/late-flowering |
| Defacio et al., 2025 | Field trial and molecular characterization | 36 | RCBD | CIMMYT/IBPGR (1991) | 15 | PCA | 4 PCs explained 75.2% variance – PC1 earliness, PC2 ear shape, PC3 yield, PC4 seed size |
| Defacio et al., 2025 | Field trial and molecular characterization | 36 | RCBD | CIMMYT/IBPGR (1991) | 15 | Pearson Correlation Analysis | Strong positive correlations: anthesis–silking (r = 0.88), plant–ear height (r = 0.87), yield–ear traits (r = 0.74–0.79) |
| Harada et al., 2009 | Field trial | 40 | Randomized grouping (12 plants/accession divided into 3 groups of 4; randomized order) | not reported | 18 | ANOVA | Significant differences (p < 0.01–0.001) for most traits; flowering and ear traits highly significant; SPAD (June 1) not significant |
| Harada et al., 2009 | Field trial | 40 | Randomized grouping (12 plants/accession divided into 3 groups of 4; randomized order) | not reported | 18 | Cluster Analysis | 4 clusters (each with 2 subclusters) grouped by earliness, ear shape, yield, and seed size; some matched known landraces |
| Harada et al., 2009 | Field trial | 40 | Randomized grouping (12 plants/accession divided into 3 groups of 4; randomized order) | not reported | 18 | PCA | 4 PCs (Eigenvalues >1) explained 75.2% total variation: PC1 = earliness of growth, PC2 = ear shape, PC3 = yield ability, PC4 = seed size |
| Kumari et al., 2017 | Field trial | 75 | Augmented Block Design | ICAR-NBPGR minimal descriptors (Mahajan et al. 2000) | 12 | ANOVA | Significant morphological differences among accessions. |
| Kumari et al., 2017 | Field trial | 75 | Augmented Block Design | ICAR-NBPGR minimal descriptors (Mahajan et al. 2000) | 12 | Cluster Analysis | 5 clusters: (I) tall/high-grain wt, (II) early/high-yield, (III–IV) medium traits, (V) late/highly prolific |
| Kumari et al., 2017 | Field trial | 75 | Augmented Block Design | ICAR-NBPGR minimal descriptors (Mahajan et al. 2000) | 12 | PCA | 3 PCs explained 75.3% variance – PC1 phenology/yield/ear traits, PC2 plant and ear height, PC3 ears per plant and grain weight |
| Sharma et al., 2010 | Field trial & Molecular characterization | 48 | Alpha Lattice | not reported | 9 | ANOVA | Significant differences observed for all traits across locations |
| Sharma et al., 2010 | Field trial & Molecular characterization | 48 | Alpha Lattice | not reported | 9 | Cluster Analysis | 4 clusters: (I) primitive late/low-yield, (II) non-primitive intermediate/good yield, (III–IV) mixed early/intermediate high-yield groups |
| Sharma et al., 2010 | Field trial & Molecular characterization | 48 | Alpha Lattice | not reported | 9 | PCA | 2 PCs explained 90% variance – PC1 (68%) yield and ear traits, PC2 (22%) kernel weight and rows |
| Belalia et al., 2019 | Field trial & Molecular characterization | 56 | Augmented Block Design | CIMMYT/IBPGR (1991) | 14 | ANOVA | Significant differences for all traits across locations |
| Belalia et al., 2019 | Field trial & Molecular characterization | 56 | Augmented Block Design | CIMMYT/IBPGR (1991) | 14 | PCA | 2 PCs explained 55.4% variance – PC1 (43%) flowering, height, ear, yield; PC2 (12%) cob diameter, ears per plant, yield |
| Rahman et al., 2015 | Field trial & Molecular characterization | 9 | RCBD | not reported | 7 | ANOVA | Significant differences (p < 0.01) among genotypes for all traits |
| Rahman et al., 2015 | Field trial & Molecular characterization | 9 | RCBD | not reported | 7 | Cluster Analysis | 3 clusters: (I) Popcorn types, (II) BHM series hybrids, (III) Sweet corn and Uttaran types |
| Rahman et al., 2015 | Field trial & Molecular characterization | 9 | RCBD | not reported | 7 | Variability Estimates | All traits highly heritable (>79%); yield per plant showed greatest variability (PCV 31.7%) |
| Dar et al., 2018 | Field trial & Molecular characterization | 50 | Not stated as standard design (evaluated in experimental plots, glasshouse + field; replicates used) | CIMMYT/IBPGR (1991) | 16 | Cluster Analysis | 3 groups identified; separation mainly by ear/cob weight, ear diameter, and stalk/leaf traits |
| Dar et al., 2018 | Field trial & Molecular characterization | 50 | Not stated as standard design (evaluated in experimental plots, glasshouse + field; replicates used) | CIMMYT/IBPGR (1991) | 16 | PCA | PC1 (41.5%) ear and cob weight; PC2 (21.1%) ear/cob diameter and leaf traits; total variance explained 86% |
| Andjelkovic et al., 2018 | Field trial & Molecular characterization | 28 | RCBD | CIMMYT/IBPGR (1991) | 21 | Cluster Analysis | 2 main groups by collection time; new accessions were taller and later flowering, older ones shorter and earlier |
| Andjelkovic et al., 2018 | Field trial & Molecular characterization | 28 | RCBD | CIMMYT/IBPGR (1991) | 21 | PCA | 5 PCs explained 80% variance; PC1 (34%) linked to plant height and ear traits; PC2 (24%) to flowering and kernel type |
| Qi-lun et al., 2008 | Field trial | 124 | RCBD | not reported | 20 | ANOVA | Only ear diameter and fresh ear weight differed significantly; other traits showed no variation. |
| Qi-lun et al., 2008 | Field trial | 124 | RCBD | not reported | 20 | Cluster Analysis | 5 main groups and 18 subgroups formed; clustering aligned with geographic origin, core set of 18 captured wide diversity |
| Kumar et al., 2015 | Field trial | 51 | Augmented Block Design | NBPGR minimal descriptors | 16 | ANOVA | Significant differences among accessions for most traits |
| Kumar et al., 2015 | Field trial | 51 | Augmented Block Design | NBPGR minimal descriptors | 16 | Cluster Analysis | 4 clusters reflecting geography and traits; Cluster 1 (J&K, HP: tall, early, high protein/sugar); Cluster 4 (Kangra: 500–1000 m asl) |
| Kumar et al., 2015 | Field trial | 51 | Augmented Block Design | NBPGR minimal descriptors | 16 | PCA | 6 PCs explained 72.6% variance – PC1 (25.4%) height, leaf, and ear traits; PC2 (15.5%); biplot aligned with cluster patterns |
| Jarić et al., 2010 | Field trial | 10 | Each accession sown in a row (single-row plots, no replication across blocks) | CIMMYT/IBPGR (1991) | 20 | ANOVA | Significant (p < 0.001) differences for most traits, especially height, leaf, ear, and kernel dimensions |
| Jarić et al., 2010 | Field trial | 10 | Each accession sown in a row (single-row plots, no replication across blocks) | CIMMYT/IBPGR (1991) | 20 | Descriptive Statistics | CV ranged 4–28%; kernel width least variable, kernel length most variable |
| Bode et al., 2012 | Field trial | 18 | Characterization field experiment (no replicated yield trial) | CIMMYT/IBPGR (1991) | 25 | Descriptive Statistics | CV 5.6–24.3%; ear height most variable; AGB1034 tallest (265 cm), AGB1032 had thick ears and heavy kernels (448.9 g) |
| Bode et al., 2012 | Field trial | 18 | Characterization field experiment (no replicated yield trial) | CIMMYT/IBPGR (1991) | 25 | Pearson Correlation Analysis | Strong positive r = 0.90 (silking–tasseling), r = 0.84 (kernels/row–ear length); negative r = −0.37 (rachis dia–kernel length) |
| Bode et al., 2014 | Field trial | 18 | Characterization field experiment (no replicated yield trial) | CIMMYT/IBPGR (1991) | 25 | Cluster Analysis | Clear grouping; AGB1026–AGB1029 closest; AGB1032 and AGB1034 distinct for size and height traits; high overall divergence |
| Carvalho et al., 2008 | Field trial | 43 | RCBD | not reported | 41 | Cluster Analysis | 4 groups: (1) tall white/high yield, (2) small white/high kernel wt, (3) short yellow/small kernels, (4) mixed types with intermediate traits |
| Carvalho et al., 2008 | Field trial | 43 | RCBD | not reported | 41 | Descriptive Statistics | Large variation in height, leaf area, ear, and kernel traits; white maize taller and more productive than yellow. |
| Carvalho et al., 2008 | Field trial | 43 | RCBD | not reported | 41 | PCA | PC1 (20.6%) and PC2 (11.1%) explained 31.8% of total variation |
| Thakur et al., 2017 | Field trial & Molecular characterization | 48 | RCBD | maize descriptors developed by the Bioversity Internationa | 15 | Cluster Analysis | 2 groups: (I) QPM/CIMMYT lines, (II) local germplasm/inbreds; indicating two gene pools |
| Thakur et al., 2017 | Field trial & Molecular characterization | 48 | RCBD | maize descriptors developed by the Bioversity Internationa | 15 | PCA | 4 PCs explained 76.9% variance – PC1 yield/height traits, PC2 kernel rows, PC3 micronutrients, PC4 protein/carotenoids |
| Thakur et al., 2017 | Field trial & Molecular characterization | 48 | RCBD | maize descriptors developed by the Bioversity Internationa | 15 | Pearson Correlation Analysis | Yield positively linked to height, cob, and grain traits; negatively with Fe, Zn, carotenoids, and kernel rows |
| Thakur et al., 2017 | Field trial & Molecular characterization | 48 | RCBD | maize descriptors developed by the Bioversity Internationa | 15 | Variability Estimates | High PCV, GCV, heritability (>90%), and genetic advance for height, cob placement, yield, and seed weight |
| Ristić et al., 2009 | Field trial & Molecular characterization | 21 | RCBD | not reported | 15 | ANOVA | Significant differences among landraces for most traits |
| Ristić et al., 2009 | Field trial & Molecular characterization | 21 | RCBD | not reported | 15 | Cluster Analysis | 2 main clusters: BD and KFD grouped together; DF types most diverse across subclusters |
| Ristić et al., 2009 | Field trial & Molecular characterization | 21 | RCBD | not reported | 15 | Descriptive Statistics | Highest CVs in tassel (20.7%) and kernel traits (15–20%); lowest in ear leaf length (5.3%) |
| Ristić et al., 2009 | Field trial & Molecular characterization | 21 | RCBD | not reported | 15 | PCA | 5 PCs explained 83.7% variance – PC1 height/leaf traits, PC2 tassel traits, PC3–PC5 kernel and ear traits |
| Ristić et al., 2014 | Field trial & Molecular characterization | 21 | RCBD | not reported | 18 | ANOVA | CV 9–29%; highest for ASI (29%), yield (23%), and ear height (22%); lowest for leaf number (9%) |
| Ristić et al., 2014 | Field trial & Molecular characterization | 21 | RCBD | not reported | 18 | Cluster Analysis | 2 main clusters with subgroups; high variability but weak separation by agro-ecological origin |
| Ristić et al., 2014 | Field trial & Molecular characterization | 21 | RCBD | not reported | 18 | Descriptive Statistics | Overall 9–29%; highest for Anthesis–silking interval (~29%), Grain yield (~23%), Ear height (~22%); lowest for Leaf number up to the ear insertion (~9%), Tassel length & Kernel thickness (~10%) |
| Ristić et al., 2014 | Field trial & Molecular characterization | 21 | RCBD | not reported | 18 | PCA | 4 PCs explained 80.9% variance – PC1 (45.9%) plant size/yield, PC2 (16.9%) tassel traits; no clear eco-group separation |
| Iqbal et al 2015 | Field trial | 153 | Augmented Block Design | CIMMYT/IBPGR (1991) | 34 | Cluster Analysis | 5 clusters at ~12.6 dissimilarity, indicating a broad genetic base |
| Iqbal et al 2015 | Field trial | 153 | Augmented Block Design | CIMMYT/IBPGR (1991) | 34 | Descriptive Statistics | High variability; greatest in yield, plant height, and 1000-kernel weight |
| Iqbal et al 2015 | Field trial | 153 | Augmented Block Design | CIMMYT/IBPGR (1991) | 34 | PCA | 7 PCs explained 70.6% variance – PC1 (26.4%) flowering traits, PC2 (12.6%) ear/cob size and kernels per row |
| Iqbal et al 2015 | Field trial | 153 | Augmented Block Design | CIMMYT/IBPGR (1991) | 34 | Pearson Correlation Analysis | Strong positive r = 0.95–0.98 among flowering traits; negative r = −0.31 (moisture–kernel wt), r = −0.29 (ASI–tassel) |
| Rivas et al 2022 | Field trial & Molecular characterization | 30 | RCBD | CIMMYT/IBPGR (1991) | 19 | Cluster Analysis | 2 groups: (1) tall, multi-ear, large-ear types; (2) short, flint/popcorn, few-leaf, long-tassel types |
| Rivas et al 2022 | Field trial & Molecular characterization | 30 | RCBD | CIMMYT/IBPGR (1991) | 19 | Descriptive Statistics | All traits varied; highest CVs in tillering (73%) and tassel traits (30–46%), lowest in leaf width and ear diameter (~8–9%) |
| Rivas et al 2022 | Field trial & Molecular characterization | 30 | RCBD | CIMMYT/IBPGR (1991) | 19 | PCA | 3 PCs explained 58.6% variance – PC1 height/tillering traits, PC2 tassel traits, PC3 minor traits |
| Rivas et al 2022 | Field trial & Molecular characterization | 30 | RCBD | CIMMYT/IBPGR (1991) | 19 | Pearson Correlation Analysis | Strong positive r = 0.87 (plant–ear height); altitude negatively correlated with leaf and kernel row number |
| Ruiz De Galarreta & Alvarez, 2001 | Field trial | 100 | RCBD | CIMMYT/IBPGR (1991) | 22 | ANOVA | Significant differences (p < 0.05) across all 22 traits in combined multi-site, multi-year analysis. |
| Ruiz De Galarreta & Alvarez, 2001 | Field trial | 100 | RCBD | CIMMYT/IBPGR (1991) | 22 | Cluster Analysis | 7 phenotypic groups with distinct plant/ear/maturity profiles |
| Ruiz De Galarreta & Alvarez, 2001 | Field trial | 100 | RCBD | CIMMYT/IBPGR (1991) | 22 | PCA | Applied to derive uncorrelated composite variables before clustering |
| Ruiz De Galarreta & Alvarez, 2001 | Field trial | 100 | RCBD | CIMMYT/IBPGR (1991) | 22 | Variability Estimates | High for cob weight (0.83), kernel rows (0.81), kernels per row (0.71), and height traits (~0.70) |
| Morales et al., 2014 | Field trial | 100 | Simple Lattice design | CIMMYT/IBPGR (1991) | 40 | ANOVA | Significant differences among populations for most traits (39/40 highly significant at P ≤ 0.01) |
| Morales et al., 2014 | Field trial | 100 | Simple Lattice design | CIMMYT/IBPGR (1991) | 40 | Cluster Analysis | 7 phenotypic groups identified with distinct plant, ear, and maturity profiles |
| Morales et al., 2014 | Field trial | 100 | Simple Lattice design | CIMMYT/IBPGR (1991) | 40 | PCA | 54.0% total (PC1=22.9%, PC2=19.8%, PC3=11.2); PC1: Days to silking, tassel branch length, no. of tassel branches, PH/EH ratio, leaf ratio, ear length/diameter; PC2: No. of grains/row, shelling factor, grain thickness/width, grain volume; PC3: Ear diameter, grain width |
| Morales et al., 2014 | Field trial | 100 | Simple Lattice design | CIMMYT/IBPGR (1991) | 40 | Pearson Correlation Analysis | Reduced 40 variables to **21** for multivariate analysis |
| Islam et al., 2020 | Field trial | 13 | RCBD | not reported | 10 | ANOVA | Significant differences among genotypes for all traits |
| Islam et al., 2020 | Field trial | 13 | RCBD | not reported | 10 | Cluster Analysis | 3 clusters; Cluster I (BHM-15, BHM-13, BHM-12, BHM-9, BHM-7) showed highest yield means |
| Islam et al., 2020 | Field trial | 13 | RCBD | not reported | 10 | PCA | 4 PCs explained 88.1% variance – PC1 (38.9%) yield and kernel traits |
| Islam et al., 2020 | Field trial | 13 | RCBD | not reported | 10 | Pearson Correlation Analysis | Yield positively correlated with plant height (r = 0.60), kernel width (0.72), 1000-kernel weight (0.81), and ear girth (0.52) |
| Islam et al., 2020 | Field trial | 13 | RCBD | not reported | 10 | Variability Estimates | High heritability for yield (96%), plant height (91%), kernels/row (87%), and 1000-kernel weight (62%) |
| Alvarado-Beltrán et al., 2019 | Field trial | 144 | Simple Lattice design | CIMMYT/IBPGR (1991) | 33 | ANOVA | 28 of 32 traits showed significant (p ≤ 0.01) genotypic differences, confirming high morphological variability |
| Alvarado-Beltrán et al., 2019 | Field trial | 144 | Simple Lattice design | CIMMYT/IBPGR (1991) | 33 | Cluster Analysis | 6 clusters; 86.6% linked to Chalqueño type, 10.4% to Cónico; variability independent of altitude |
| Alvarado-Beltrán et al., 2019 | Field trial | 144 | Simple Lattice design | CIMMYT/IBPGR (1991) | 33 | Pearson Correlation Analysis | Highly correlated traits removed; 16 key traits retained for further diversity analysis |
| N’da et al., 2015 | Field trial | 118 | Simple Lattice design | CIMMYT/IBPGR (1991) | 22 | ANOVA | Significant group differences; silking, anthesis, ear, and plant height most discriminating |
| N’da et al., 2015 | Field trial | 118 | Simple Lattice design | CIMMYT/IBPGR (1991) | 22 | Cluster Analysis | 5 groups (r = 0.72); Group 3 largest with superior yield traits |
| N’da et al., 2015 | Field trial | 118 | Simple Lattice design | CIMMYT/IBPGR (1991) | 22 | Descriptive Statistics | Wide variability across 22 traits; anthesis 50–74 days, plant height 175–306 cm, kernel weight 21–37 g |
| N’da et al., 2015 | Field trial | 118 | Simple Lattice design | CIMMYT/IBPGR (1991) | 22 | PCA | 3 PCs explained 84.6% variance – PC1 precocity/vegetative traits, PC2 yield, PC3 kernel rows |
| Kasoma et al., 2021 | Field trial and molecular characterization | 59 | Alpha lattice | not reported | 13 | ANOVA | Significant (p < 0.001) variation for flowering, height, ear, yield, and FAW resistance traits. |
| Kasoma et al., 2021 | Field trial and molecular characterization | 59 | Alpha lattice | not reported | 13 | Cluster Analysis | 2 clusters; related landraces clustered by province |
| Kasoma et al., 2021 | Field trial and molecular characterization | 59 | Alpha lattice | not reported | 13 | PCA | 2 PCs captured major variation; yield and FAW resistance traits loaded on different axes. |
| Kasoma et al., 2021 | Field trial and molecular characterization | 59 | Alpha lattice | not reported | 13 | Pearson Correlation Analysis | Height and flowering traits positively correlated; FAW damage negatively associated with yield |
| Kasoma et al., 2021 | Field trial and molecular characterization | 59 | Alpha lattice | not reported | 13 | Variability Estimates | Heritability ranged 0.24–0.93; yield and flowering traits showed high (>0.50) values |
| Kumar et al., 2021 | Field trial | 39 | RCBD | not reported | 12 | ANOVA | Significant differences; high heritability for seed weight, ear diameter, height traits; yield strongly environment-influenced. |
| Kumar et al., 2021 | Field trial | 39 | RCBD | not reported | 12 | Cluster Analysis | 7 clusters identified; clusters V and VI contained high-yielding genotypes (e.g., MZM-44, MZM-34, MZM-37, MZM-23) |
| Kumar et al., 2021 | Field trial | 39 | RCBD | not reported | 12 | PCA | 5PCs explained **73.6%** of total variation; PC1 driven by test weight, yield per plant, ear diameter, and plant height |
| Kumar et al., 2021 | Field trial | 39 | RCBD | not reported | 12 | Pearson Correlation Analysis | Yield correlated positively with ear diameter (r = 0.24) and seed weight (r = 0.48), negatively with flowering time |
| Kumar et al., 2021 | Field trial | 39 | RCBD | not reported | 12 | Variability Estimates | High heritability and genetic advance for 1000-seed weight, ear diameter, and plant height, indicating additive gene action and selection potential. Least environmental influence for flowering traits (small GCV–PCV gap) |
| Del Carmen Flores-Rosales et al., 2015 | Field trial | 76 | Simple Lattice design | CIMMYT/IBPGR (1991) | 7 | ANOVA | Significant differences detected across all traits |
| Del Carmen Flores-Rosales et al., 2015 | Field trial | 76 | Simple Lattice design | CIMMYT/IBPGR (1991) | 7 | Cluster Analysis | Populations grouped by region; Tehuacan accessions had highest cornhusk values—suggested as selection criteria |
| Ferro et al., 2007 | Field trial | 85 | Split–split plot | not reported | 9 | ANOVA | Significant differences among maturity groups and within landraces; strong G×E effects, no landrace × N interaction |
| Ferro et al., 2007 | Field trial | 85 | Split–split plot | not reported | 9 | Pearson Correlation Analysis | Positive correlations among GDU, plant size, and yield; lodging negatively related; kernel weight independent |
| Ferro et al., 2007 | Field trial | 85 | Split–split plot | not reported | 9 | Variability Estimates | Moderate–high heritability (0.59–0.84) for morphology, low for yield (0.35–0.39); strong genetic correlations across N levels |
| Gouesnard et al., 2016 | Field trial (managed stress environment) | 78 | Alpha Lattice | not reported | 13 | Pearson Correlation Analysis | Yield showed strong positive correlation with kernels per plot |
| Gouesnard et al., 2016 | Field trial (managed stress environment) | 78 | Alpha Lattice | not reported | 13 | Variability Estimates | Large genetic variability; yield and kernel traits declined under drought stress |
| Hong et al., 2024 | 1 (field trial) + historical RDA data reports | 50 | No design (Single-site validation field trial (2023) | not reported | 6 | Correlation Analysis with RDA dataset (scatter plots) | Field trial results aligned closely with RDA dataset trends |
| Hong et al., 2024 | Field trial | 50 | No design | not reported | 35 | PCA | PC1 and PC2, which explain 36.78 % and 27.63 % of the variance, respectively (biomass-related traits and flowering time) |
| Hong et al., 2024 | Field trial | 50 | No design | not reported | 35 | Pearson Correlation Analysis | Strong correlations among flowering time, plant architecture (ear height, plant height, and leaf length), and ear traits (ear weight, ear length, and weight of kernels per ear) |
| Balconi et al., 2024 | field trial (multilocation) and molecular | 588 | No design (Multi-environment, randomized, standardized EVA protocol (not a strict RCBD or lattice—varied across locations)) | not reported | 6 | ANOVA | Significant difference detected across environments for all traits |
| Balconi et al., 2024 | field trial (multilocation) and molecular | 588 | No design (Multi-environment, randomized, standardized EVA protocol (not a strict RCBD or lattice—varied across locations)) | not reported | 6 | Cluster Analysis | 9 groups identified; clustering aligned with geography and genetic structure |
| Balconi et al., 2024 | field trial (multilocation) and molecular | 588 | No design (Multi-environment, randomized, standardized EVA protocol (not a strict RCBD or lattice—varied across locations)) | not reported | 6 | PCA | Major PCs reflected variability in flowering and plant architecture traits |
| Babić et al., 2021 | field trial | 298 | RCBD | CIMMYT/IBPGR (1991) | 26 | Cluster Analysis | 5 clusters detected; redundancy estimated at ~27.5% (white) and ~25.7% (yellow-orange) |
| Hartings et al., 2008 | Field trial & Molecular characterization | 54 | RCBD | CIMMYT/IBPGR (1991) | 20 | ANOVA | Significant differences among landraces; high heritability for most traits |
| Hartings et al., 2008 | Field trial & Molecular characterization | 54 | RCBD | CIMMYT/IBPGR (1991) | 20 | Cluster Analysis | UPGMA grouped 54 landraces into 4 clusters based on ear type and geographic origin |
| Hartings et al., 2008 | Field trial & Molecular characterization | 54 | RCBD | CIMMYT/IBPGR (1991) | 20 | PCA | 5 PCs explained 86.3% variance – PC1 (56.1%) earliness/size, PC2 (10.6%) kernel qualit |
| Nelimor et al., 2019 | Field trial | 36 | Alpha Lattice | CIMMYT/IBPGR (1991) | 16 | ANOVA | Significant year, genotype, and G×Y effects for yield and most traits under all stress conditions; genotypes contributed largest variation; high repeatability |
| Nelimor et al., 2019 | Field trial | 36 | Alpha Lattice | CIMMYT/IBPGR (1991) | 16 | Cluster Analysis | Groups separated by stress tolerance: 5 clusters (MDS), 2 (HS), 3 (DSHS) |
| Nelimor et al., 2019 | Field trial | 36 | Alpha Lattice | CIMMYT/IBPGR (1991) | 16 | Correlation Analysis (Genotypic & Phenotypic) | Strong yield correlation HS–DSHS (r ≈ 0.94), moderate OGC–MDS (≈0.65), weak MDS–DSHS (≈−0.01); flowering traits consistently positive |
| Nelimor et al., 2019 | Field trial | 36 | Alpha Lattice | CIMMYT/IBPGR (1991) | 16 | Variability Estimates | Grain yield heritability ranged 0.37–0.87; most traits moderately to highly repeatable (>0.60) |
| Pressoir & Berthaud, 2004 | Field trial and molecular characterization | 31 | Two-replicate hierarchical design (population & family plots) | CIMMYT/IBPGR (1991) | 15 | ANOVA | Significant population differentiation observed across trait |
| Pressoir & Berthaud, 2004 | Field trial and molecular characterization | 31 | Two-replicate hierarchical design (population & family plots) | CIMMYT/IBPGR (1991) | 15 | Cluster Analysis | Populations clustered differently under phenotypic vs molecular analyses |
| Pressoir & Berthaud, 2004 | Field trial and molecular characterization | 31 | Two-replicate hierarchical design (population & family plots) | CIMMYT/IBPGR (1991) | 15 | Variability Estimates | Moderate–high variability; flowering traits likely overestimated due to assortative mating |
| Toxtle-Flores et al., 2023 | Field trial | 64 | Simple Lattice design | not reported | 24 | ANOVA | Significant (p < 0.0001) differences across 37 traits; stability observed with minimal G×E interaction |
| Toxtle-Flores et al., 2023 | Field trial | 64 | Simple Lattice design | not reported | 24 | Cluster Analysis | 64 accessions grouped into 4 clusters; grain color and earliness key discriminators; most aligned with Chalqueño race |
| Toxtle-Flores et al., 2023 | Field trial | 64 | Simple Lattice design | not reported | 24 | PCA | PC1 leaf area, PC2 leaf and kernel traits, PC3 ear and tassel traits; confirmed clear diversity structure |
| Toxtle-Flores et al., 2023 | Field trial | 64 | Simple Lattice design | not reported | 24 | Pearson Correlation Analysis | 37 traits reduced to 27 after removing highly correlated variables (r ≥ \|0.7\|). |
| Goyanka et al., 2021 | Field trial | 99 | Augmented Block Design | Bioversity International descriptors for maize genetic resources (Alercia, 2011) | 30 | ANOVA | Significant differences for all traits except ear width and ears per plant. |
| Goyanka et al., 2021 | Field trial | 99 | Augmented Block Design | Bioversity International descriptors for maize genetic resources (Alercia, 2011) | 30 | Cluster Analysis | 99 accessions grouped into 8 clusters; largest contained 27 accessions |
| Goyanka et al., 2021 | Field trial | 99 | Augmented Block Design | Bioversity International descriptors for maize genetic resources (Alercia, 2011) | 30 | Descriptive statistics | Broad variation across 14 traits; plant height 115–253 cm, tasseling 43–89 days |
| Goyanka et al., 2021 | Field trial | 99 | Augmented Block Design | Bioversity International descriptors for maize genetic resources (Alercia, 2011) | 30 | PCA | PC1 = 78.25% (plant height, ear height, ear length, tassel branching); PC2 = 9.99% (flowering traits); PC3 = 5.59% (leaf traits, 100-seed weight) |
| Goyanka et al., 2021 | Field trial | 99 | Augmented Block Design | Bioversity International descriptors for maize genetic resources (Alercia, 2011) | 30 | Pearson Correlation Analysis | Strong r = 0.99 (tasseling–silking), r = 0.90 (plant–ear height); flowering negatively correlated with kernels/row (r ≈ –0.42) |
| Goyanka et al., 2021 | Field trial | 99 | Augmented Block Design | Bioversity International descriptors for maize genetic resources (Alercia, 2011) | 30 | Variability Estimates | High heritability (>50%) for flowering, height, ear, and kernel traits |
| Ilarslan et al., 2002 | Field trial | 32 | RCBD | CIMMYT/IBPGR (1991) | 25 | ANOVA | Significant differences among races and accessions; accession variance large (17–83%) |
| Ilarslan et al., 2002 | Field trial | 32 | RCBD | CIMMYT/IBPGR (1991) | 25 | Pearson Correlation Analysis | Strong positive associations among kernel, ear, and vegetative traits; negative with rachis segment length |
| Ilarslan et al., 2002 | Field trial | 32 | RCBD | CIMMYT/IBPGR (1991) | 25 | Variability Estimates | Accession-level variance exceeded race-level (e.g., tassel length 82.75%, kernel length 17.17%) |
| Salazar et al., 2017 | Field trial | 34 | RCBD | CIMMYT/IBPGR (1991) | 41 | Cluster Analysis | 34 accessions grouped into 4 clusters with clear subgrouping |
| Salazar et al., 2017 | Field trial | 34 | RCBD | CIMMYT/IBPGR (1991) | 41 | Descriptive Statistics | Showed wide variability among traits; CV up to 48% in tassel and ear traits |
| Salazar et al., 2017 | Field trial | 34 | RCBD | CIMMYT/IBPGR (1991) | 41 | PCA | First 3 PCs explained 73% of variation; PC1 = architecture + precocity; PC2 = ear traits; PC3 = tassel traits |
| Salazar et al., 2017 | Field trial | 34 | RCBD | CIMMYT/IBPGR (1991) | 41 | Pearson Correlation Analysis | Ear height (EH), Plant height (PH), and Ear leaf length (ELL) highly correlated (r > 0.9); weak correlations between vegetative and ear/kernel traits |
| Singode et al., 2009 | Field trial | 57 | RCBD | not reported | 7 | ANOVA | Significant differences among accessions for yield traits at both sites; ear length significant at 5%, others at 1% |
| Wei et al., 2009 | Field trial and molecular characterization | 104 | RCBD | not reported | 12 | ANOVA | Significant differences among landraces for all key traits (p < 0.01); high phenotypic variability |
| Wei et al., 2009 | Field trial and molecular characterization | 104 | RCBD | not reported | 12 | Cluster Analysis | Cluster analysis grouped 102 landraces into 2–3 major groups; some homonymous landraces distinct |
| Wei et al., 2009 | Field trial and molecular characterization | 104 | RCBD | not reported | 12 | PCA | PCA captured major variation in yield and morphological traits, separating landraces by performance |
