# Supplementary figures and images for: Phenotypic Diversity in Maize Landraces: A Systematic Review of Global Patterns, Methodological Approaches, and Implications for Breeding
Source: Genes (Basel). 2026 Mar 31;17(4):413. doi: 10.3390/genes17040413 (PMC13115568; doi:10.3390/genes17040413)

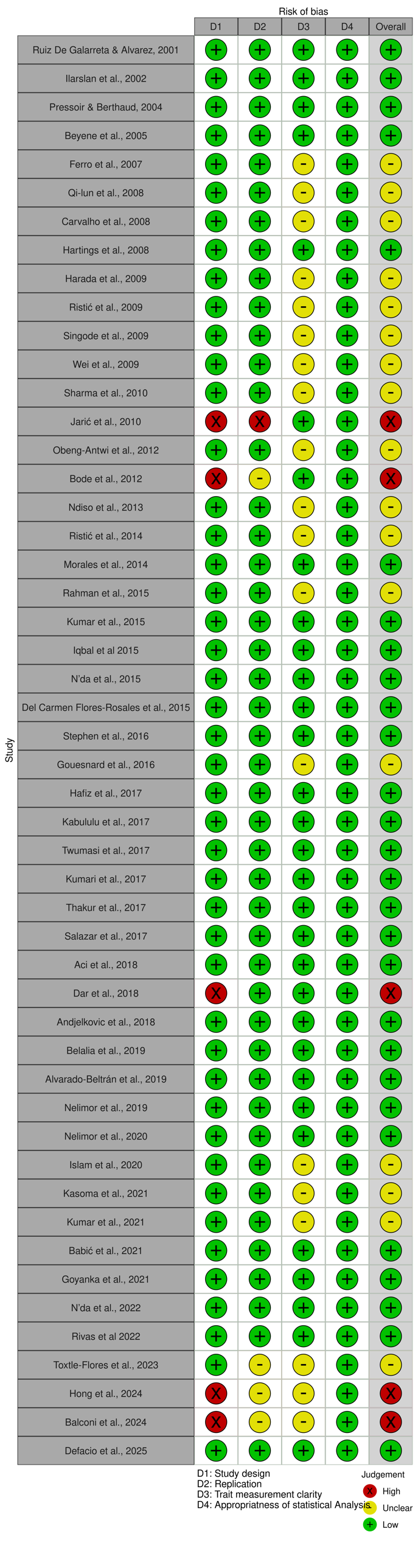

Supplement: Supplementary file 1 [file genes-17-00413-s001.zip › Figure S1. presents the risk-of-bias traffic light plots for all 50 included studies.png]
